# A genome-wide association study reveals specific transferases as candidate loci for bovine milk oligosaccharides synthesis

**DOI:** 10.1186/s12864-019-5786-y

**Published:** 2019-05-22

**Authors:** Nina A. Poulsen, Randall C. Robinson, Daniela Barile, Lotte B. Larsen, Bart Buitenhuis

**Affiliations:** 1 Department of Food Science, Aarhus University, Blichers Allé 20, P. O. Box 50, DK-8830 Tjele, Denmark; 2 Department of Food Science and Technology, University of California, Davis, One Shields Ave., Davis, CA 95616 USA; 3 Foods for Health Institute, University of California, Davis, One Shields Ave., Davis, CA 95616 USA; 4 Center for Quantitative Genetics and Genomics, Department of Molecular Biology and Genetics, Aarhus University, Blichers Allé 20, P. O. Box 50, DK-8830 Tjele, Denmark

**Keywords:** Oligosaccharides, GWAS, Candidate genes, Milk

## Abstract

**Background:**

Human milk oligosaccharides (OS) play a key role in brain and gut microbiota development of the neonate, but the underlying biosynthetic steps of OS in the mammary gland are still largely unknown. As bovine milk contains OS with somewhat similar structures and functionalities there is increased interest in further understanding the genetic basis underlying the OS content of milk for eventual extraction and generation of value-added ingredients for infant formulas and nutraceuticals. The present study is the first to report on genetic parameter estimation as well as on a genome wide association study (GWAS) from the largest bovine milk OS dataset analyzed to date.

**Results:**

In total 15 different bovine milk OS were monitored. Heritabilities ranged from 0 to 0.68 in Danish Holstein and from 0 to 0.92 in Danish Jersey. The GWAS identified in total 1770 SNPs (FDR < 0.10) for five different OS in Danish Holstein and 6913 SNPs (FDR < 0.10) for 11 OS in Danish Jersey. In Danish Holstein, a major overlapping QTL was identified on BTA1 for LNH and LNT explaining 24% of the variation in these OS. The most significant SNPs were associated with *B3GNT5*, a gene encoding a glycosyltransferase involved in glycan synthesis. In Danish Jersey, a very strong QTL was detected for the OS with composition 2 Hex 1 HexNAc (isomer 1) on BTA11. The most significant SNP had –log_10_(*P*-value) of 52.88 (BOVINEHD1100030300) and was assigned to *ABO*, a gene encoding ABO blood group glycosyltransferases. This SNP has been reported to be a missense mutation and explains 56% of the OS variation. Other candidate genes of interest identified for milk OS were *ALG3, B3GALNT2, LOC520336*, *PIGV, MAN1C1, ST6GALNAC6, GLT6D1, GALNT14, GALNT17*, *COLGALT2*, *LFNG* and *SIGLEC.*

**Conclusion:**

To our knowledge, this is the first study documenting a solid breeding potential for bovine milk OS and a strong indication of specific candidate genes related to OS synthesis underlying this genetic influence. This new information has the potential to guide breeding strategies to achieve production of milk with higher diversity and concentration of OS and ultimately facilitate large-scale extraction of bovine milk OS.

**Electronic supplementary material:**

The online version of this article (10.1186/s12864-019-5786-y) contains supplementary material, which is available to authorized users.

## Background

Free oligosaccharides (OS) are bioactive molecules present in human milk that provide numerous health benefits to developing infants, including stimulating growth of selected beneficial bacteria in the gut, participating in development of the brain and exerting anti-pathogenic activity by preventing pathogen binding to intestinal epithelial cells [1, 2]. The underlying biosynthetic steps of OS in the mammary gland are still largely unknown, but genes encoding glycosyltransferases, glycosidases and sugar transporters are expected to play a role. In light of such numerous health benefits attributed to these molecules, recent studies have set out to characterize the structures and bioactivities of free bovine OS in milk and in dairy streams, and further to develop processing techniques that would allow these molecules to be isolated and used as food ingredients [3–6]. Despite some differences in abundance, structural complexity, and diversity between human and bovine OS, bovine milk contains several OS structures in common with human milk [3, 7]. Furthermore, their utility as health-promoting bioactive compounds has been demonstrated in a recent study showing improvements in metabolism and physical development when animal models of infant under-nutrition were fed diets supplemented with bovine milk OS [4]. Given the vast amount of whey originating from cheese production, recovery and up-concentration of bovine milk OS from dairy streams could be a valuable source of OS for use as bioactive ingredients, especially for the purposes of enhancing the functionalities of infant formula and developing value-added ingredients for nutraceutical applications [8–10].

Bovine milk OS are generally smaller in size than those of human milk, with less complex structures and fewer isomers for each composition [11]. Milk OS are synthesized from glucose (Glc), galactose (Gal), N-acetylglucosamine (GlcNAc), N-acetylgalactosamine (GalNAc), fucose (Fuc), N-acetylneuraminic acid (NeuAc) and N-glycolylneuraminic acid (NeuGc) [12], likely by the action of specific glycosyltransferases [13]. Early studies on bovine OS focused on bovine colostrum and early lactation milk [11, 14, 15]. Studies on mature bovine milk documented decreased OS abundance from early to mid-lactation, and showed that acidic (sialic acid-containing) OS decreased more rapidly compared to the neutral OS [16]. In a small set of milk samples from Danish Holstein (DH) and Danish Jersey (DJ) cattle, differences in OS abundances were found between breeds [17]. OS in milk from DJ cows contained higher relative amounts of both sialylated and the more complex neutral fucosylated OS, whereas milk from DH had a higher abundance of smaller and simpler neutral OS [17]. That study also revealed that bovine milk contains several larger fucosylated structures (containing up to 10 monosaccharide units) and not just simple OS as previously thought.

Wickramasinghe et al. [13] examined expression of glycosylation-related genes by sequencing of RNA extracted from milk somatic cells at day 15 and 250 in lactation from three Holstein and three Jersey cows. They revealed no significant difference in expressions of glycosylation-related genes between the two breeds. However, out of 121 genes examined, 92 were expressed in the milk somatic cells and most of these exhibited higher expression at 250 days of milking compared with early lactation [13]. They found 29 genes to be important for the synthesis of sialylated OS, and 70 genes of importance for the synthesis of fucosylated OS, suggesting that the abundances of both of these OS classes are genetically influenced. The use of genome wide association studies (GWAS) to explore genetic influence underlying the variation in bovine milk OS has, to our knowledge, not been documented before. This most likely relates to the need for highly advanced analytical techniques that are both high-throughput and sensitive enough for quantifying low-abundance bovine milk OS. We have used isobaric tags for an optimized mass spectrometry-based OS quantification method [18], which has enabled relative OS quantification in more than 600 milk samples from DH and DJ [19]. The results confirmed that DJ milk contains higher amounts of most bovine OS. In both breeds, variation in OS abundance was strongly affected by parity [19]. The aim of the present study was to estimate genetic parameters and conduct a GWAS in order to examine whether OS in DH and DJ milk are heritable and to identify underlying single nucleotide polymorphism (SNP) markers affecting OS variation in bovine dairy breeds.

## Results

### Variation in oligosaccharides between breeds

In Table 1, the relative phenotypic mean, standard deviations and CVs for the OS in milk of DH and DJ measured by Nano-LC Chip Q-ToF are presented. The OS are represented by their monosaccharide compositions, denoted as the quantity of each monosaccharide type present in the structure (Hexose (Hex)_N-acetylhexosamine (HexNAc)_Fuc_NeuAc_NeuGc). None of the identified structures included NeuGc. DJ milk contained significantly greater abundance, on average, of most OS. Phenotypic correlations were pronounced for some of the OS. In particular, the OS with compositions 3 Hex 2 HexNAc and 4 Hex 1 HexNAc were strongly positively correlated within each breed (*r* = 0.91 for DH and *r* = 0.89 for DJ). Further, Lacto-N-hexaose (LNH) and Lacto-N-tetraose (LNT) were strongly positively correlated especially in DJ (*r* = 0.55 for DH and *r* = 0.90 for DJ) and the four fucosylated OS were strongly positively correlated in DJ, though not in DH.

**Table 1:** Mean and standard deviation of oligosaccharide abundances in milk from Danish Holstein and Danish Jersey

Trait^a,b^	Holstein			Jersey		
	Mean	SD	CV	Mean	SD	CV
2_0_0_1_0 (3′-sialyllactose)	0.788	0.280	35.5%	1.300	0.478	36.8%
2_0_0_1_0 (6′-sialyllactose)	0.716	0.326	45.6%	1.351	0.539	39.9%
2_0_0_2_0	1.046	0.410	39.2%	1.681	0.735	43.7%
2_1_0_0_0 isomer 1	1.406	1.211	86.1%	1.481	1.414	95.5%
2_1_0_0_0 isomer 2	1.320	0.490	37.1%	1.300	0.526	40.5%
3_1_0_0_0 (Lacto-N-tetraose)	0.938	0.302	32.2%	1.686	1.133	67.2%
3_1_0_0_0 Isomer 2	0.527	0.295	56.1%	1.134	0.403	35.6%
3_2_0_0_0	0.787	0.612	77.8%	1.835	1.407	76.6%
3_6_1_0_0	0.577	0.208	36.1%	1.267	1.065	84.1%
4_1_0_0_0	0.932	0.590	63.3%	2.198	1.193	54.2%
4_2_0_0_0 (Lacto-N-hexaose)	0.495	0.195	39.4%	1.609	1.408	87.5%
4_4_1_0_0	0.706	0.304	43.1%	1.294	0.649	50.1%
4_5_1_0_0	0.775	0.354	45.7%	1.664	1.917	115.2%
5_4_0_0_0	0.705	0.426	60.5%	0.383	0.261	68.2%
5_4_1_0_0	0.845	0.319	37.7%	1.215	0.722	59.4%

^a^Oligosaccharides are represented by their monosaccharide compositions, denoted as Hex_HexNAc_Fuc_NeuAc_NeuGc

^b^OS abundance values are expressed as the mass spectral intensity of the isobaric label reporter ions relative to that of a spiked internal standard of the same parent mass

### Heritabilities

The heritability estimates and the genetic variance for 15 individual OS for DH and DJ are presented in Table 2. In DH, high heritabilities were found for 4 Hex 1 HexNAc (0.68), 3 Hex 2 HexNAc (0.67), 2 Hex 1 HexNAc isomer 1 (structure: GalNac(α1–3) Gal(β1–4)Glc, 0.65), 3 Hex 6 HexNAc 1 Fuc (0.57), 5 Hex 4 HexNAc 1 Fuc (0.54), LNH (0.52) and 5 Hex 4 HexNAc (0.47). For DJ, high heritabilities were found for LNH (0.92), LNT (0.79), 2 Hex 1 HexNAc isomer 1 (0.74), 4 Hex 1 HexNAc (0.63), 5 Hex 4 HexNAc 1 Fuc (0.58), 3 Hex 6 HexNAc 1 Fuc (0.55), 3 Hex 1 HexNAc isomer 2 (0.47) and 5 Hex 4 HexNAc (0.42). The heritability estimates for 2 Hex 2 NeuAc, 2 Hex 1 HexNAc isomer 2, and 4 Hex 4 HexNAc 1 Fuc were not significantly different from zero in both breeds. This was also the case for 6′-sialyllactose (6′-SL) in DH. Generally, the acidic OS (containing NeuAc) had low to insignificant heritabilities in both breeds.

**Table 2:** Additive genetic variance (σ^2^_A_), and heritability (h^2^) (means±SE) for 15 individual oligosaccharides in Danish Holstein (DH) and Danish Jersey (DJ)

Trait^a^	DH σ^2^_A_	DH h^2^	DJ σ^2^_A_	DJ h^2^
2_0_0_1_0	0.014 (0.12)	0.25 (0.18)	0.040 (0.20)	0.20 (0.18)
2_0_0_1_0	0.003 (0.06)	0.03 (0.13)	0.067 (0.26)	0.24 (0.19)
2_0_0_2_0	2.21E-07 (0.00)	1.04E-06 (0.11)	0.018 (0.14)	0.03 (0.17)
2_1_0_0_0 isomer 1	0.956 (0.98)	0.65 (0.24)	1.57 (1.25)	0.74 (0.24)
2_1_0_0_0 isomer 2	0.059 (0.24)	0.13 (0.15)	1.08E-07 (0.00)	3.81E-07 (0.13)
3_1_0_0_0 LNT	0.024 (0.16)	0.27 (0.18)	1.144 (1.07)	0.79 (0.26)
3_1_0_0_0 isomer 2	0.017 (0.13)	0.23 (0.18)	0.087 (0.30)	0.47 (0.22)
3_2_0_0_0	0.244 (0.49)	0.67 (0.25)	0.419 (0.65)	0.22 (0.17)
4_1_0_0_0	0.233 (0.48)	0.68 (0.25)	0.910 (0.95)	0.63 (0.23)
4_2_0_0_0 LNH	0.020 (0.14)	0.52 (0.21)	2.087 (1.44)	0.92 (0.26)
5_4_0_0_0	0.087 (0.30)	0.47 (0.25)	0.032 (0.18)	0.42 (0.23)
3_6_1_0_0	0.024 (0.16)	0.57 (0.22)	0.681 (0.83)	0.55 (0.23)
4_4_1_0_0	1.65E-07 (0.00)	1.05E-06 (0.14)	0.122 (0.35)	0.19 (0.19)
4_5_1_0_0	0.027 (0.16)	0.20 (0.19)	0.880 (0.94)	0.23 (0.19)
5_4_1_0_0	0.054 (0.23)	0.54 (0.21)	0.347 (0.59)	0.58 (0.24)

^a^Oligosaccharides are represented by their monosaccharide compositions, denoted as Hex_HexNAc_Fuc_NeuAc_NeuGc. (SE of the estimates in parenthesis)

### GWAS

The entire set of GWAS results are presented in Additional file 1: Table S1 for DH and Additional file 2: Table S2 for DJ together with QQ plots for the individual OS (Additional file 3: Figure S3 for DH and Additional file 4: Figure S4 for DJ). The files include the allele-substitution effect, location and annotation. In total, 1770 significant SNPs (FDR < 0.10) were detected for five OS in DH and 6913 significant SNPs (FDR < 0.10) for 11 OS in DJ. For the three acidic OS (3′-sialyllactose (3′-SL), 6′-SL and 2 Hex 2 NeuAc), no significant SNPs were found in DH nor in DJ. Among the non-fucosylated neutral OS, no significant SNPs were found in either breed for 3 Hex 1 HexNAc isomer 2. Further, there were no significant SNPs for the 2 Hex 1 HexNAc isomers or 3 Hex 2 HexNAc in DH. For the fucosylated OS, no significant SNPs were found for 3 Hex 6 HexNAc 1 Fuc, 4 Hex 4 HexNAc 1 Fuc, or 4 Hex 5 HexNAc 1 Fuc in DH. The significant SNPs were scattered at many chromosomes (Additional file 1: Table S1 and Additional file 2: Table S2). The number of SNPs assigned to candidate genes for OS synthesis, as well as their significance (*p*-value) and percentage of explained phenotypic variance are presented in Table 3 for DH and Table 4 for DJ.

**Table 3:** Significant SNPs assigned to candidate genes for oligosaccharide synthesis in Danish Holstein

Gene	Gene name	Chr.^b^	# SNP^c^	Highest -log_10_(*P*-value)	PVE%^d^	Trait^a^	
B3GNT5	UDP-GlcNAc:betaGal beta-1.3-N-acetylglucosaminyltransferase 5	1	4	20.779	24.02	3_1_0_0_0 LNT	
B3GNT5	UDP-GlcNAc:betaGal beta-1.3-N-acetylglucosaminyltransferase 5	1	3	19.771	22.94	4_2_0_0_0 LNH	
LOC520336	N-acetyllactosaminide beta-1.6-N-acetylglucosaminyl-transferase. Isoform C	23	2	6.318	7.34	4_2_0_0_0 LNH	
ALG3	ALG3. alpha-1.3- mannosyltransferase	1	1	6.307	7.32	4_2_0_0_0 LNH	
ALG3	ALG3. alpha-1.3- mannosyltransferase	1	1	5.054	5.76	3_1_0_0_0 LNT	
B3GALNT2	beta-1.3-N-acetylgalactosaminyltransferase 2	28	2	5.539	6.37	5_4_0_0_0	
GLT6D1	glycosyltransferase 6 domain containing 1	11	2	5.124	5.85	4_1_0_0_0	

^a^Oligosaccharides are represented by their monosaccharide compositions, denoted as Hex_HexNAc_Fuc_NeuAc_NeuGc

^b^*Chr* chromosome

^c^*#SNP* number of significant SNPs assigned to candidate gene

^d^*PVE%* Percentage of phenotypic variation explained

**Table 4:** Significant SNPs assigned to candidate genes for oligosaccharide synthesis in Danish Jersey

Gene	Gene name	Chr.^b^	#SNP^c^	Highest -log_10_(*P*-value)	PVE%^d^	Trait^a^
ABO	ABO blood group (transferase *A. alpha* 1–3-N-acetylgalactosaminyltransferase; transferase *B. alpha* 1–3-galactosyltransferase)	11	19	52.879	55.87	2_1_0_0_0 isomer 1
GALNT17	polypeptide N-acetylgalactosaminyltransferase 17 like	25	28	27.690	33.99	3_6_1_0_0
GALNT17	polypeptide N-acetylgalactosaminyltransferase 17 like	25	26	23.712	29.74	5_4_1_0_0
GALNT17	polypeptide N-acetylgalactosaminyltransferase 17 like	25	18	20.326	25.92	4_5_1_0_0
GALNT17	polypeptide N-acetylgalactosaminyltransferase 17 like	25	12	14.215	18.55	4_4_1_0_0
GALNT17	polypeptide N-acetylgalactosaminyltransferase 17 like	25	15	13.206	17.28	5_4_0_0_0
MAN1C1	mannosidase alpha class 1C member 1	2	6	6.677	8.64	3_1_0_0_0 LNT
MAN1C1	mannosidase alpha class 1C member 1	2	6	5.654	7.23	4_2_0_0_0 LNH
ST6GALNAC6	ST6 N-acetylgalactosaminide alpha-2.6-sialyltransferase 6	11	1	6.599	8.53	2_1_0_0_0 isomer 1
GLT6D1	glycosyltransferase 6 domain containing 1	11	2	5.556	7.10	2_1_0_0_0 isomer 1
PIGV	phosphatidylinositol glycan anchor biosynthesis class V	2	2	5.534	7.07	4_2_0_0_0 LNH
PIGV	phosphatidylinositol glycan anchor biosynthesis class V	2	1	5.328	6.78	3_1_0_0_0 LNT
LFNG	LFNG O-fucosylpeptide 3-beta-N-acetylglucosaminyltransferase	25	1	4.763	6.00	5_4_1_0_0
LFNG	LFNG O-fucosylpeptide 3-beta-N-acetylglucosaminyltransferase	25	1	4.523	5.67	4_4_1_0_0
GALNT14	polypeptide N-acetylgalactosaminyltransferase 14	11	3	3.979	4.91	3_1_0_0_0 LNT
COLGALT2	collagen beta(1-O)galactosyltransferase 2	16	2	3.906	4.81	4_1_0_0_0
SIGLEC1	sialic acid binding Ig like lectin 1	13	1	3.850	4.73	3_6_1_0_0

^a^Oligosaccharides are represented by their monosaccharide compositions, denoted as Hex_HexNAc_Fuc_NeuAc_NeuGc

^b^*Chr* chromosome

^c^*#SNP* number of significant SNPs assigned to candidate gene

^d^*PVE%* Percentage of phenotypic variation explained

### Danish Holstein

For the non-fucosylated neutral OS, a total of 1755 significant SNPs were detected including 390 for LNT, 112 for 4 Hex 1 HexNAc, 736 for LNH and 517 for 5 Hex 4 HexNAc (Additional file 1: Table S1). A number of significant SNPs were shared among the non-fucosylated neutral OS within DH. This includes 226 significant SNPs shared for LNT and LNH, three between 5 Hex 4 HexNAc and LNT, and 10 between 4 Hex 1 HexNAc and 5 Hex 4 HexNAc (data not shown). The most significant SNP (ARS-BFGL-NGS-31112) in DH was detected on BTA10 for 5 Hex 4 HexNAc with a –log_10_(*P*-value) of 21.63. This SNP was not assigned to any gene. In total 19 SNPs were significant for 5 Hex 4 HexNAc on BTA10, but none were assigned to genes for OS synthesis.

**Fig. 1:**
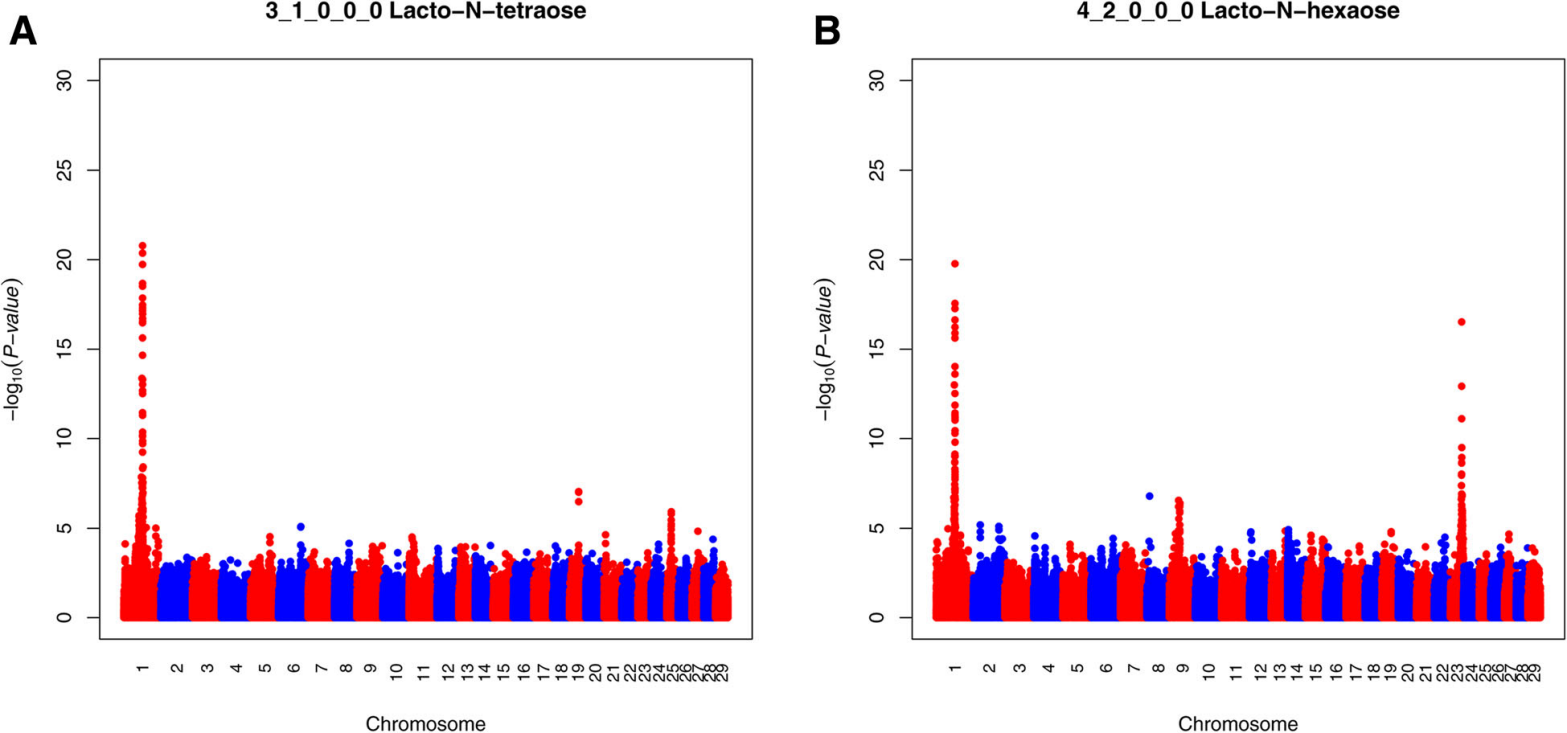
Manhattan plot for LNT and LNH in Danish Holstein. The Y-axes show the –log_10_ (*P*-value) of single-marker association tests. The X-axes show marker positions in base-pairs

For LNT and LNH a strong QTL peak was detected on BTA1, where the most significant SNP marker for LNT was BOVINEHD0100024184, with a –log_10_(*P*-value) of 20.78, and the most significant SNP for LNH was BOVINEHD0100024179 with a –log_10_(P-value) of 19.77. Both these SNPs were assigned to *B3GNT5*, which encodes UDP-GlcNAc:β-Gal β-1-3-N-acetylglucosaminyltransferase 5 (Fig. 1, Table 3). The three top SNPs were shared for LNT and LNH (BOVINEHD0100024179; BOVINEHD0100024181; BOVINEHD0100024184), all assigned to *B3GNT5*. Further, the SNP BTB-01702174, also assigned to *B3GNT5*, was significant for LNT (−log_10_(*P*-value) of 18.69). The top SNPs explained 24% of the phenotypic variance for LNT and 23% of the phenotypic variance for LNH (Table 3). In total, 289 and 340 significant SNPs were detected on BTA1 for LNH and LNT, respectively, and all of the shared significant SNPs between LNT and LNH were distributed on BTA1. Apart from *B3GNT5*, another shared significant SNP (BOVINEHD0100023936) was found on BTA1 with a –log_10_(*P*-value) of 6.31 for LNH and a –log_10_(P-value) of 5.05 for LNT, respectively. This SNP was assigned to *ALG3*, a gene encoding α-1-3-mannosyltransferase and explained 7.3% of the phenotypic variance for LNH and 5.8% for LNT (Table 3). Aside from these candidate genes, the BTA1 QTL contained a number of other genes that might also be of interest (Additional file 1 Table S1) despite their lack of obvious association to OS variability.

For LNH, a strong QTL was also detected on BTA23 (Fig. 1) and the most significant SNP had a –log_10_(*P*-value) of 16.53 (BOVINEHD2300013179). This SNP was not assigned to any gene, but the region contained two significant SNPs assigned to *LOC520336* (BOVINEHD2300013151; BOVINEHD2300013152) with –log_10_(*P*-value) of 6.32 and 6.03, respectively. This gene encodes N-acetyllactosaminide β-1-6-N-acetylglucosaminyl-transferase, isoform C and explained 7.3% of the phenotypic variance for LNH. Other candidate genes of interest include *B3GALNT2* on BTA28 for 5 Hex 4 HexNAc, which encodes β-1-3-N-acetylgalactosaminyltransferase 2. In total, 11 SNPs were significant for 5 Hex 4 HexNAc on BTA28, and the two most significant SNPs (BOVINEHD2800013694; BOVINEHD4100018494), which both had a –log_10_(*P*-value) of 5.54, were assigned to *B3GALNT2* and explained 6.4% of the phenotypic variance (Table 3).

For 4 Hex 1 HexNAc, 112 significant SNPs were distributed over 18 chromosomes with no clear QTLs. The most significant SNP was on BTA1 (BOVINEHD0100014437) with a –log_10_(*P*-value) of 8.82. This SNP was not assigned to any gene. The 10 significant SNPs for 4 Hex 1 HexNAc on BTA1 did not overlap with the common QTL for LNT and LNH, and none of the SNPs could be assigned to a gene. Fifteen significant SNPs were identified on BTA11, and the two most significant SNPs (BOVINEHD1100030076; BOVINEHD1100030077), which both had –log_10_(*P*-value) of 5.12, were assigned to *GLT6D1*, a gene encoding glycosyltransferase 6 domain containing 1. Together these SNPs explained 5.9% of the phenotypic variance (Table 3). Further, two SNPs on BTA11 were assigned to *PAEP*, which is the gene encoding progestagen-associated endometrial protein (β-lactoglobulin). None of the 10 overlapping SNPs common between 4 Hex 1 HexNAc and 5 Hex 4 HexNAc could be assigned to any gene, but seven of these were located on BTA1. The three common SNPs between 5 Hex 4 HexNAc and LNT were all located on BTA25, but could not be assigned to any gene.

For the fucosylated neutral OS 5 Hex 4 HexNAc 1 Fuc, 15 significant SNPs were detected (Additional file 1: Table S1). Thirteen of the significant SNPs were located on BTA22 and the most significant SNP had a –log_10_(*P*-value) of 7.29, but could not be assigned to any gene.

### Danish Jersey

**Fig. 2:**
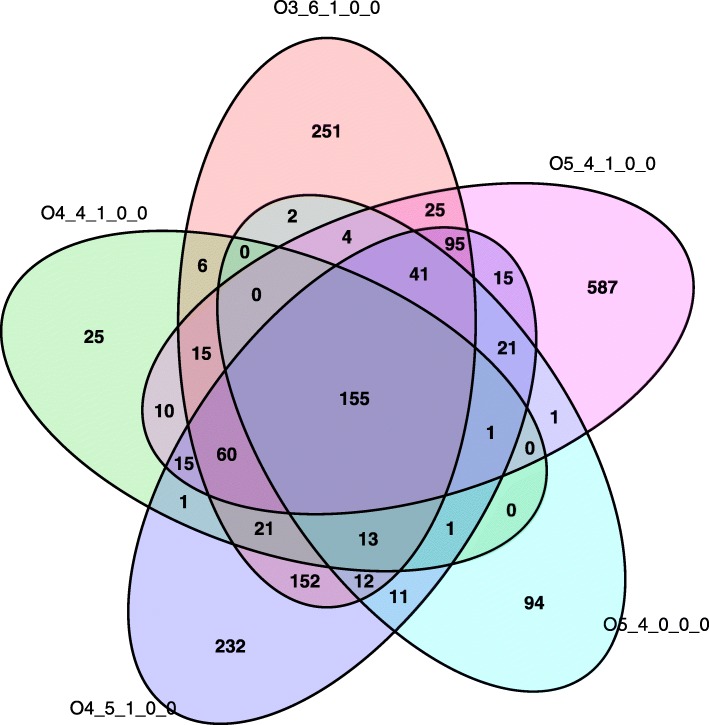
Shared significant SNPs for four fucosylated oligosaccharides and one non-fucosylated neutral oligosaccharide in Danish Jersey. Numbers in Venn diagram refers to number of significant SNPs shared. Oligosaccharides are represented by their monosaccharide compositions, denoted as Hex_HexNAc_Fuc_NeuAc_NeuGc

For the non-fucosylated neutral OS, a total of 3847 significant SNPs were detected for 2 Hex 1 HexNAc isomer 1 (691), 2 Hex 1 HexNAc isomer 2 (16), LNT (1039), 3 Hex 2 HexNAc (122), 4 Hex 1 HecNAc (623), LNH (1000) and 5 Hex 4 HexNAc (356) (Additional file 2 Table S2). For the fucosylated OS, a total of 3066 significant SNPs were detected for 3 Hex 6 HexNAc 1 Fuc (852), 4 Hex 4 HexNAc 1 Fuc (323), 4 Hex 5 HexNAc 1 Fuc (846) and 5 Hex 4 HexNAc 1 Fuc (1045). In total, 880 significant SNPs were shared between LNT and LNH. Further, a very high number of significant SNPs were shared among the fucosylated OS as well as among the fucosylated OS and the neutral OS 5 Hex 4 HexNAc (Venn diagram, Fig. 2). Apart from these, a single significant SNP on BTA2 (BOVINEHD0200033124) was shared between LNT, LNH and 3 Hex 6 HexNAc 1 Fuc. This SNP could not be assigned to any gene.

**Fig. 3:**
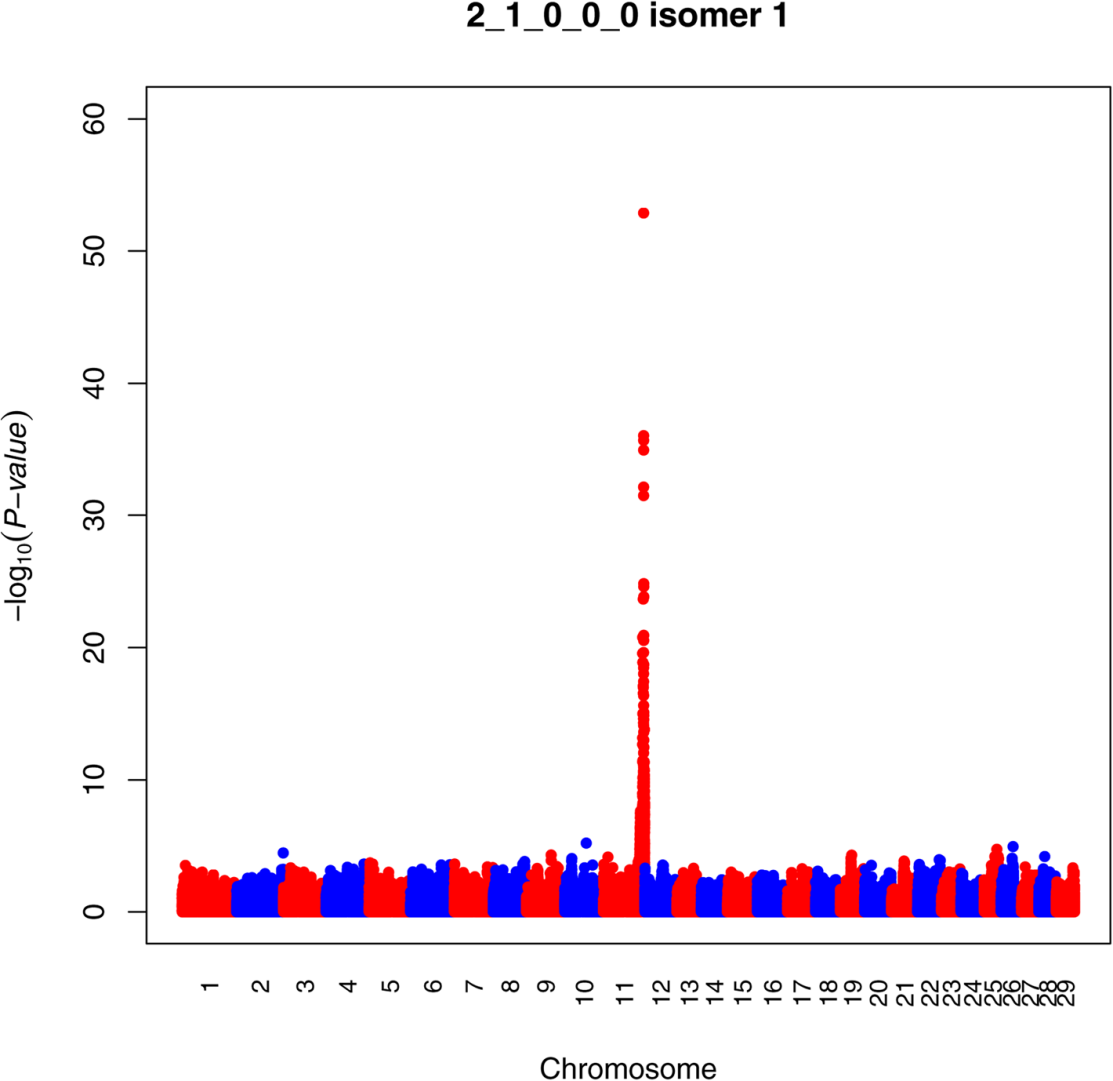
Manhattan plot for 2 Hex 1 HexNAc isomer 1 in Danish Jersey. The Y-axes show the –log_10_(*P*-value) of single-marker association tests. The X-axes show marker positions in base-pairs

A very strong QTL was detected for 2 Hex 1 HexNAc isomer 1 on BTA11 (Fig. 3). The most significant SNP had –log_10_(*P*-value) of 52.88 (BOVINEHD1100030300) and explained 56% of the trait variance. This SNP was assigned to *ABO*, a gene which encodes the ABO blood group (transferase A. α-1-3-N-acetylgalactosaminyltransferase; transferase B. α-1-3-galactosyltransferase). In total, 19 SNPs within this QTL were assigned to the gene with –log_10_(*P*-value) ranging from 9.98 to 52.88. The BTA11 QTL for 2 Hex 1 HexNAc isomer 1 also contained significant SNPs assigned to two other candidate genes (*ST6GALNAC6* and *GLT6D1*) in addition to numerous other genes (Table 4, Additional file 2: Table S2). One significant SNP (BOVINEHD1100028669) with a –log_10_(*P*-value) of 6.60 was assigned to *ST6GALNAC6*, a gene encoding ST6 N-acetylgalactosaminide α-2-6-sialyltransferase 6 explaining 8.5% of the trait variance (Table 4). Further, two significant SNPs (BOVINEHD1100030074, BOVINEHD1100030075), which had a –log_10_(*P*-value) of 5.56 and 4.56, respectively, were assigned to *GLT6D1*, a gene encoding glycosyltransferase 6 domain containing 1. Together these explained 7.1% of the phenotypic variance. In addition, one SNP on BTA11 was assigned to *PAEP*. For 2 Hex 1 HexNAc isomer 2 only 16 significant SNPs were detected, all located on BTA1, but with no assignment to any known genes.

**Fig. 4:**
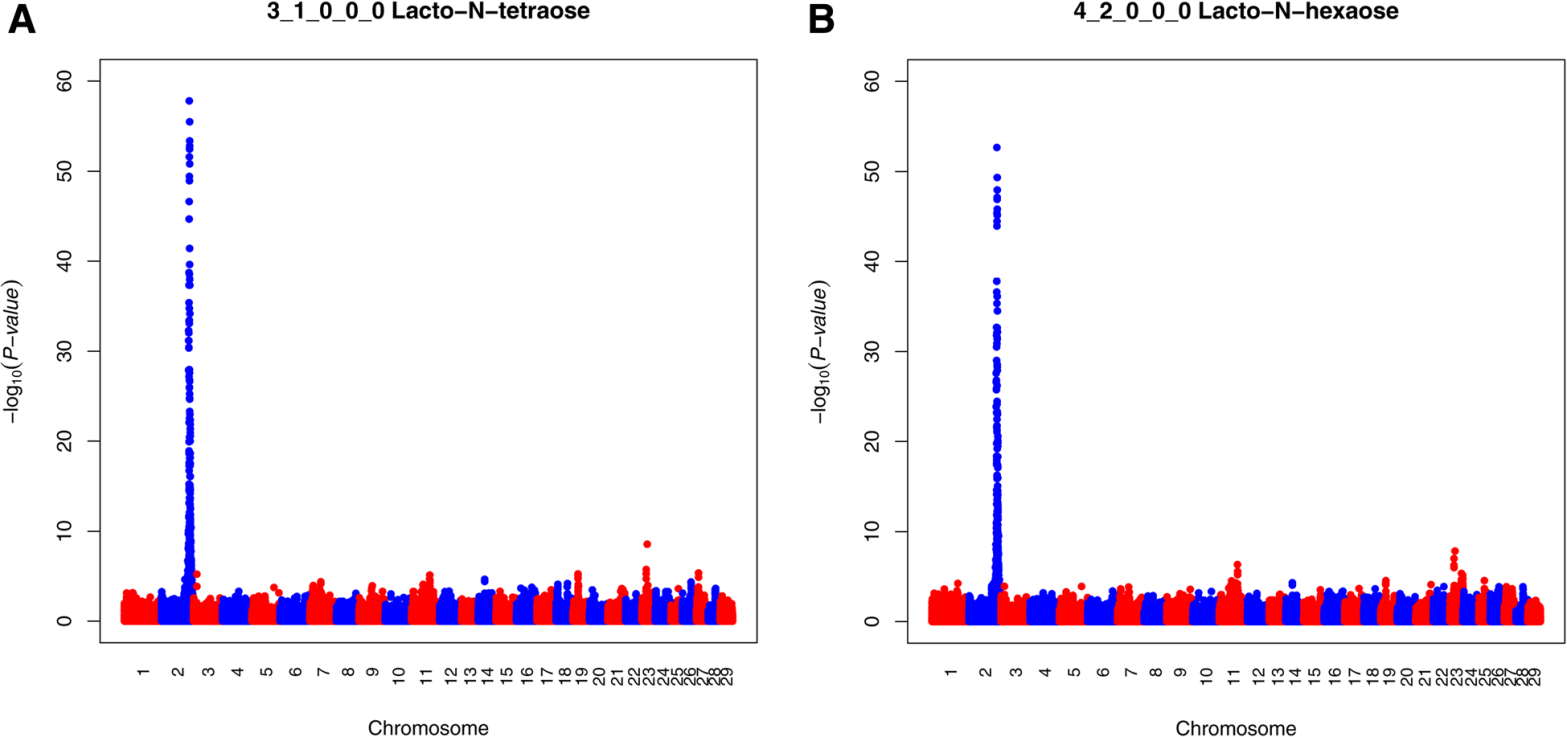
Manhattan plot for LNT and LNH in Danish Jersey. The Y-axes show the –log_10_(*P*-value) of single-marker association tests. The X-axes show marker positions in base-pairs

In total, 848 of the 880 significant SNPs common for LNT and LNH were located within a very strong QTL on BTA2 (Fig. 4). The most significant SNP for LNT explained 59% of the variation and had a –log_10_(*P*-value) of 57.84 (BOVINEHD0200034794), while for LNH the most significant SNP had a –log(*P*-value) of 52.68 (BOVINEHD0200034794), explaining 56% of the trait variance. This SNP could not be assigned to any gene. The QTL contains numerous genes, including two candidate genes related to OS synthesis (*PIGV* and *MAN1C1*; Table 4). Six shared significant SNPs for LNT and LNH were assigned to *MAN1C1* (BOVINEHD0200037177, BOVINEHD0200037178, BOVINEHD0200037168, BOVINEHD0200037167, BOVINEHD0200037173, BOVINEHD0200037175). The most significant of these SNPs was BOVINEHD0200037177 in both traits with –log_10_(*P*-value) of 6.68 for LNT and 5.65 for LNH, respectively. This gene encodes mannosidase alpha class 1C member 1 and explained 8.6% of the phenotypic variance for LNT and 7.2% of the phenotypic variance for LNH (Table 4). Furthermore, two significant SNPs for LNH were assigned to *PIGV* (BOVINEHD0200036859, BOVINEHD0200036858), a gene encoding phosphatidylinositol glycan anchor biosynthesis class V. These SNPs had –log_10_(*P*-value) of 5.53 and 4.45, respectively. BOVINEHD0200036859 was also significant for LNT with a –log_10_(*P*-value) of 5.33 (Table 4). These SNPs explained 7% of the phenotypic variance for LNH and 6.8% of the phenotypic variance for LNT (Table 3). Apart from the BTA2 QTL and related genes, three significant SNPs (BOVINEHD1100019501, BOVINEHD1100019509, BOVINEHD1100019520) for LNT on BTA11 were assigned to *GALNT14*, a gene encoding polypeptide N-acetylgalactosaminyltransferase 14 (Table 4). These SNPs had –log_10_(*P*-value) of 3.98, 3.75 and 3.75, respectively and explained 4.9% of the phenotypic variance.

For 4 Hex 1 HexNAc in DJ, a strong QTL was found on BTA16, and the most significant SNP had –log_10_(*P-*value) of 20.35 (BOVINEHD1600014578) and was assigned to *AGRN*, a gene encoding agrin (Additional file 2 Table S2). Another significant SNP was also assigned to this gene (BOVINEHD1600014580) and had –log_10_(*P*-value) of 10.42. The 122 significant SNPs for 3 Hex 2 HexNAc were scattered over 13 chromosomes, but 57 SNPs were overlapping with 4 Hex 1 HexNAc, and likewise the most significant SNP for this OS was BOVINEHD1600014578 with a –log_10_(*P*-value) of 9.75. For 4 Hex 1 HexNAc, two significant SNPs (BOVINEHD1600018857, BOVINEHD1600018860) were assigned to the candidate gene *COLGALT2* within the BTA16 QTL, which encodes collagen beta(1-O)galactosyltransferase 2. These both had a –log_10_(*P*-value) of 3.91 and explained 4.8% of the trait variance.

The non-fucosylated neutral OS 5 Hex 4 HexNAc was strongly associated with the fucosylated neutral OS, as revealed by the high number of shared significant SNPs (Fig. 2). In total, 155 significant SNPs were shared among the four fucosylated OS (3 Hex 6 HexNAc 1 Fuc, 4 Hex 4 HexNAc 1 Fuc, 4 Hex 5 HexNAc 1 Fuc and 5 Hex 4 HexNAc 1 Fuc) and 5 Hex 4 HexNAc. Further, 152 significant SNPs were unique for 3 Hex 6 HexNAc 1 Fuc and 4 Hex 5 HexNAc 1 Fuc, 95 were unique for 3 Hex 6 HexNAc 1 Fuc, 4 Hex 5 HexNAc 1 Fuc and 5 Hex 4 HexNAc 1 Fuc, and 60 significant SNPs were unique for the four fucosylated OS. A strong QTL was detected on BTA25 with 423 significant SNPs shared for 3 Hex 6 HexNAc 1 Fuc and 4 Hex 5 HexNAc 1 Fuc. The most significant SNP within this QTL was BOVINEHD4100017380 for 3 Hex 6 HexNAc 1 Fuc and 4 Hex 5 HexNAc 1 Fuc with –log_10_(*P*-value) of 31.65 and 22.51, respectively. Likewise, 4 Hex 4 HexNAc 1 Fuc, 5 Hex 4 HexNAc, and 5 Hex 4 HexNAc 1 Fuc also had a strong QTL on BTA25 and a high number of shared significant SNPs with the other fucosylated OS. BOVINEHD4100017380 was the most significant SNP for 4 Hex 4 HexNAc 1 Fuc, 5 Hex 4 HexNAc, and 5 Hex 4 HexNAc 1 Fuc with –log_10_(*P*-value) of 16.79, 13.44 and 24.06, respectively. This SNP could not be assigned to any gene. However, for the four fucosylated OS and 5 Hex 4 HexNAc a high number of significant SNPs on BTA25 were assigned to *WBSCR17* alias *GALNT17*), which encodes polypeptide N-acetylgalactosaminyltransferase 17 like. The most significant SNP assigned to this gene was BOVINEHD2500008220 for 3 Hex 6 HexNAc 1 Fuc, 5 Hex 4 HexNAc 1 Fuc, 4 Hex 5 HexNAc 1 Fuc, 4 Hex 4 HexNAc 1 Fuc, and 5 Hex 4 HexNAc with –log_10_(P-value) of 27.69, 23.71, 20.33, 14.22 and 13.21, respectively (Table 4). SNPs assigned to GALNT17 explained from 17 to 34% of the phenotypic variance (Table 4). Furthermore, for 4 Hex 4 HexNAc 1 Fuc and 5 Hex 4 HexNAc 1 Fuc a significant SNP (ARS-BFGL-NGS-65615) on BTA25 was assigned to *LFNG*, a candidate gene encoding O-fucosylpeptide 3-β-N-acetylglucosaminyltransferase (Table 4). This SNP explained 6% of the trait variance for 5 Hex 4 HexNAc 1 Fuc and 5.7% for 4 Hex 4 HexNAc 1 Fuc. Another candidate gene was identified on BTA 13 for 3 Hex 6 HexNAc 1 Fuc as one significant SNP (BOVINEHD1300014785) with –log_10_(*P*-value) of 3.85 was assigned to *SIGLEC1*, which encodes sialic acid binding Ig like lectin 1 (Table 4). This SNP explained 4.7% of the phenotypic variance (Table 4). Furthermore, in total 98 significant SNPs were detected on BTA13 for 3 Hex 6 HexNAc 1 Fuc, but no specific candidate genes were identified (Additional file 2 Table S2).

### Overlapping SNPs of Danish Jersey and Danish Holstein

The GWAS revealed a large number of significant SNP in DH and DJ, and strong genetic association between specific OS within breeds. However, only 11 significant SNPs were common for DH and DJ. In both breeds, significant SNPs were assigned to the candidate gene *GLT6D1,* with two significant SNPs (BOVINEHD1100030074, BOVINEHD1100030075) assigned to *GLT6D1* for 2 Hex 1 HexNAc isomer 1 in DJ and two significant SNPs (BOVINEHD1100030076; BOVINEHD1100030077) assigned to *GLT6D1* for 4 Hex 1 HexNAc in DH. As denoted, any OS, which were below the level of quantification had zeroes entered for the genetic analysis. In DH, four OS had zeroes in more than 10 milk samples, while this was the case for three OS in DJ. This slightly violated assumptions of normal distribution. However, no significant SNPs were identified in the GWAS and low to non-existing h^2^ were estimated for the OS in DH. Likewise, two of the OS had low to non-existing h^2^ in DJ, whereas for 5 Hex 4 HexNAc, where 15 samples were denoted with zero values h^2^ was 0.42 (SE 0.23).

### Functional importance of candidate genes

**Fig. 5:**
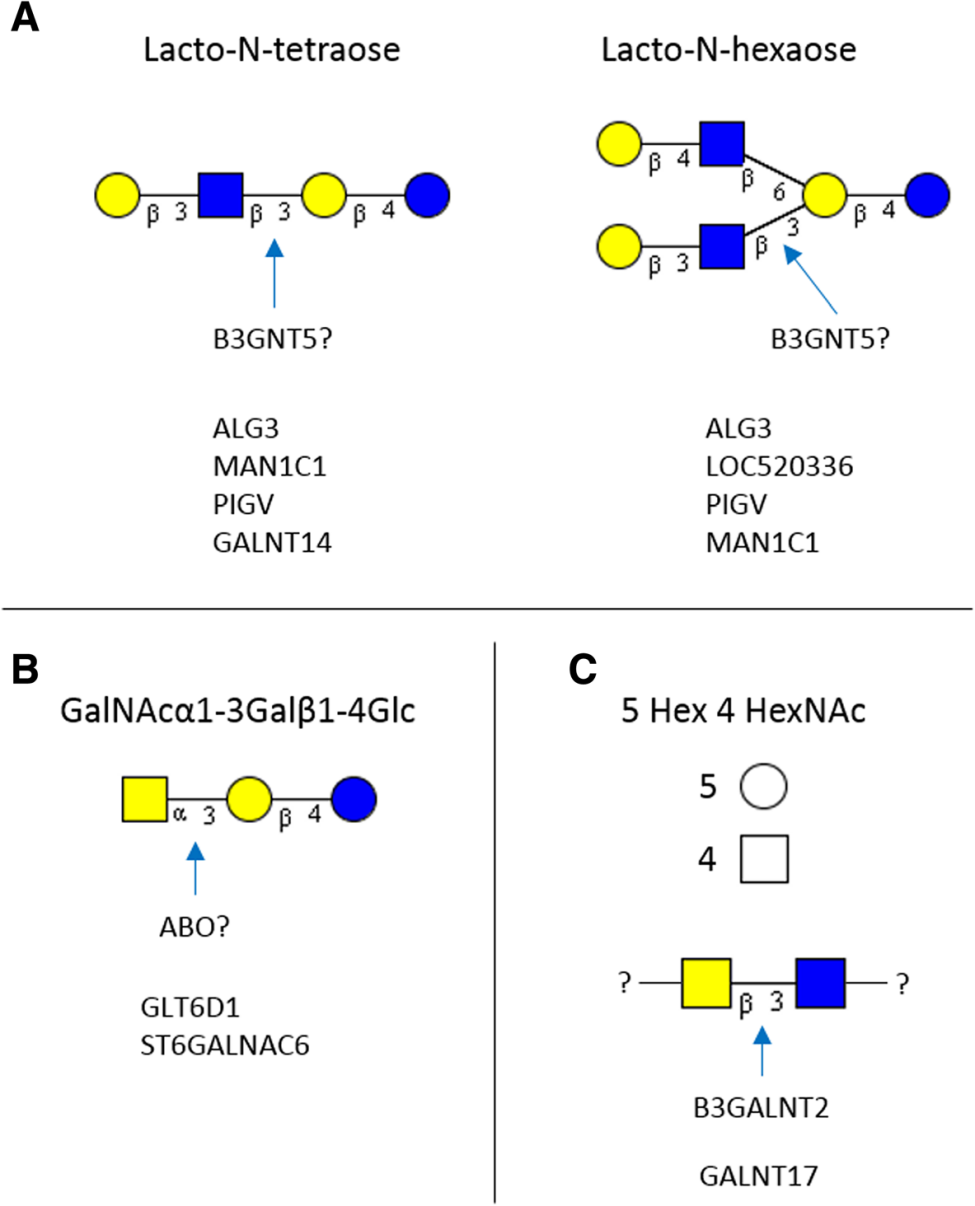
Association between oligosaccharides transferases identified as candidate genes and their speculated role on biosynthesis of LNT, LNH, 2 Hex 1 HexNAc and 5 Hex 4 HexNAc

Significant SNPs assigned to candidate genes for OS synthesis were checked to see whether any of the SNPs were associated with interesting protein sequence variation. Most of the markers were assigned to intergenic or intronic regions, but the SNP with the highest –log_10_(*P*-value) in the full data set was reported as a missense mutation. This is the SNP (BOVINEHD1100030300), which is significant for 2 Hex 1 HexNAc isomer 1 in DJ and is assigned to the *ABO* gene. The most promising candidate genes and their role for synthesis of specific OS in the mammary gland are illustrated in Fig. 5.

## Discussion

Advanced analytical techniques in combination with efficient labeling methods enabling sample multiplexing have ensured high-quality OS profiling data in the largest dataset reported to date. The phenotype evaluated was the relative content of each OS compared among the milk samples. In the current study, all cows were in mid-lactation and these results are therefore translatable to commercially available milk.

### Heritabilities for bovine oligosaccharides

To our knowledge, this is the first study to estimate heritabilities for OS in bovine milk. Seven OS in DH and eight in DJ had moderate to high heritabilities (h^2^ > 0.4). For some OS the same trend was found in DH and DJ: most notably the low to insignificant heritabilities for the acidic OS were evident in both breeds. This suggests that the content of sialylated OS cannot be improved through selective breeding. However, this result does not represent a concern because bovine milk already contains a high proportion of sialylated OS (70% in colostrum and 50% in mature milk, [16]), while fucosylated OS abundance is rather low. To better mimic the composition of human milk, increasing the content of more complex and lower-abundance fucosylated OS should be of interest. Several of the neutral non-fucosylated OS (including LNT and LNH), as well as fucosylated neutral OS (especially 3 Hex 6 HexNAc 1 Fuc and 5 Hex 4 HexNAc 1 Fuc), display moderate to high heritabilities, suggesting a good potential to increase the content of these specific OS and improve the commercial sourcing potential of bovine milk. The higher content of OS in DJ milk and higher heritabilities for some OS compared to DH suggests that DJ cows may be the breed of choice for customizing OS production such that the OS ensemble more closely resembles human milk.

### QTL and candidate genes for oligosaccharides

Free milk OS synthesis is very complex: a literature review has identified more than 121 glycosylation-related genes which included several glycosyltransferases, glycosidases and glycan transporters [13]. The concerted action of various glycosyltransferases is likely needed for OS synthesis. In human and bovine milk, most OS contain lactose at the reducing end, which can be elongated with lacto-N-biose or N-acetyllactosamine and further sialylated or fucosylated at the terminal end [3, 20]. It has been postulated that milk OS are formed through modification of lactose by glycosyltransferases [20], but the specific enzymatic pathways are still largely uncharacterized. Transcriptome profiling of bovine milk OS has confirmed expression of several glycosylation-related genes in RNA extracted from milk somatic cells [13], which provides a list of possible candidate genes. These studies in conjunction with GWAS could substantially contribute to further elucidating OS synthetic pathways. In the present study, the number of significant SNPs were almost fourfold higher in DJ than in DH. For both breeds, the low heritabilities for acidic OS were reflected in a lack of significant SNPs detected, suggesting very limited genetic influence on the variation of these OS. In contrast, 2 Hex 1 HexNAc isomer 1, 3 Hex 2 HexNAc, 3 Hex 6 HexNAc 1 Fuc, and 5 Hex 4 HexNAc 1 Fuc show moderate to high heritabilities. These OS have no or very few significant SNPs detected in DH, probably indicating a more polygenic influence of these specific OS. The results in DJ indicate that the genetic background for the fucosylated OS are under the same regulation with a high number of significant SNPs shared. As this was also true for 5 Hex 4 HexNAc, this could indicate that this relatively complex OS is dependent on some of the same pathways during synthesis, as a positive rather than a negative correlation was observed between 5 Hex 4 HexNAc and the fucosylated OS.

### *B3GNT5* in Danish Holstein

The most promising QTL in DH is on BTA1 and identified for LNT and LNH. The three top SNPs were shared for both OS and were assigned to *B3GNT5*. This gene encodes a specific glycosyltransferase, β3-N-acetylglucosaminyltransferases, which is an enzyme catalyzing the elongation of a glycan by adding a β-N-acetylglucosamine residue at the C3 position of galactose [21]. This feature is present in both LNT and LNH, and the gene is therefore a very strong candidate gene for the synthesis of these OS structures. The significant SNPs for both OS assigned to *B3GNT5* are all listed as intronic variants, and a deeper exploration of the region and the traits association is thus needed to establish the direct relationship. The gene was found only to be weakly expressed in the study by [13].

### Other candidate genes in Danish Holstein

Apart from *B3GNT5* in DH, several other less significant candidate genes were also detected (Table 3). For LNT and LNH, significant SNPs were assigned to the *ALG3* gene within the same QTL as *B3GNT5* on BTA1. The gene encodes α-1,3-mannosyltransferase, which is an enzyme responsible for the addition of mannose in an α-1,3-linkage to GlcNAc. Mannose has not been identified in free milk OS, but in N-linked glycans, where the glycan chain is covalently bound to asparagine residues, the basic core structure is rich in mannose [1] and ALG3 plays a central role in the synthesis of the N-linked core moiety [22]. Thus, *ALG3* is not expected to play a role for free OS in milk, unless there is a stronger connection identified between the synthesis of free and bound glycans in milk. For LNH, significant SNPs on BTA23 were assigned to *LOC520336*, which is most likely a gene encoding another glycosyltransferase, N-acetyllactosaminide-1,6-N-acetylglucosaminyltransferase. This enzyme specifically catalyzes the bonding of N-acetylglucosamine to the Gal of N-acetyllactosamine in a 1,6 linkage. LNT is a linear oligosaccharide having only 1 N-acetylglucosamine attached, in comparison to the branched LNH containing 2 N-acetylglucosamine residues. However, as the additional N-acetylglucosamine in LNH is attached to Gal of the lactose, and not to the N-acetyllactosamine, the association to this enzyme is not clear. For 5 Hex 4 HexNAc, significant SNPs assigned to the gene *B3GALNT* on BTA 28 were detected. This gene encodes another glycosyltransferase, β-1,3-N-acetylgalactosaminyltranseferase. This suggests that at least one of the 4 N-acetylhexosamines within the structure of this OS is an N-acetylgalactosamine. For 4 Hex 1 HexNAc, significant SNPs assigned to the gene *GLT6G1* on BTA11 were detected. This gene encodes another glycosyltransferase gene family related to the ABO blood type system, and will be discussed in more detail for DJ below, where *ABO* is identified as a candidate gene.

### ABO in Danish Jersey

In DJ, a major QTL was identified on BTA11 for 2 Hex 1 HexNAc isomer 1 and had 19 SNPs assigned to the *ABO* gene. This gene is involved in the ABO blood type system. In cattle, the blood type system is more complex and includes more factors than those observed in humans [23]. In humans, variation in the gene affects the expression of different transferases. Type A individuals express α-1,3-N-acetylgalactosaminyltransferase, type B individuals express α-1,3-galactosyltransferase, type AB individuals express both of these, and type O individuals express neither [24]. The action of these two glycosyltransferases results in the transformation of the H antigen into A or B antigens [24]. The molecular background in humans is related to a few SNPs changing four amino acid residues in the transferase, whereas a single base deletion results in the inactive gene defining type O(H) individuals [24]. The ABO system in humans relates to the secretor system, as the A and B transferases can only function when at least one α-1,2-fucosyltransferase is active [25]. The bovine ABH blood type system may be different from the human ABO and have other substrate specificity [25]. The OS 2 Hex 1 HexNAc isomer 1 does not contain fucose, which may suggest that the action of the ABO gene is not dependent on the same substrate as in humans. The structural analysis of the 2 Hex 1 HexNAc isomer 1 revealed an α-1,3 linkage of N-acetylgalactosamine to lactose [26], which indicates action of transferase A. The most significant SNP assigned to the ABO gene was reported as a missense mutation, which potentially could affect the protein function directly. It is very rare for a GWAS study in cattle to identify strong candidate genes within QTLs and even rarer that the study would identify genetic variation potentially affecting the trait of interest through changed protein functions [27]. Further studies should enable establishment of a direct link between the mutation and variation in 2 Hex 1 HexNAc isomer 1. The paralogous *GLT6D1* gene is located in very close proximity to the *ABO* gene on BTA11 [25]. This gene encodes another glycosyltransferase, with a yet unknown catalytic specificity. However, the gene and its location in close proximity to the *ABO* gene is highly conserved across species [25], and, interestingly, it was detected as a candidate gene in both DH and DJ. In a study using the same DH samples from the current study for a GWAS of milk metabolites, a significant QTL for glucose on BTA11 was located between *LOC100848307* and *ABO* [28], suggesting some association between free glucose in milk and ABO. This may indicate an association between the levels of free monosaccharides and free oligosaccharides in milk. In close proximity to this major QTL on BTA11, significant SNPs were assigned to the gene *ST6GALNAC6*. This gene encodes ST6 N-acetylgalactosaminide α-2,6-sialyltransferase 6, an enzyme belonging to sialyltransferase family, which probably catalyses the addition of sialic acid to a N-acetylgalactosamine through an α-2,6 linkage. As 2 Hex 1 HexNAc is not sialylated, the association could relate to enzymes competing for the same substrate (e.g. lactose or more complex OS), and a lower activity of *ST6GALNAC6* could thus lead to increased content of 2 Hex 1 HexNAc. Expression of *ST6GALNAC6* has been reported earlier in Jersey and Holstein cows, but only *ST6GALNAC2* and *ST6GALNAC5* displayed significant change in expression from early to mid-lactation [13].

### Candidate genes for fucosylated OS in Danish Jersey

For the fucosylated OS a high number of significant SNPs were detected. The most significant SNPs were on BTA25 and a high number of these were assigned to *GALNT17*, which most likely encodes polypeptide N-acetylgalactosaminyltransferase 17. Apart from the fucosylated OS, significant SNPs for 5 Hex 4 HexNAc were also assigned to this gene. Compared to human milk OS, where the only HexNAc moiety is N-acetylglucosamine [20], bovine milk OS also contains N-acetylgalactosamine [3, 29] and the association between complex OS and *GALNT17* in the current study could indicate that that the elongation of more complex OS structures in bovine milk are related to N-acetylgalactosamine. Furthermore, for 4 Hex 4 HexNAc 1 Fuc and 5 Hex 4 HexNAc 1 Fuc another significant SNP on BTA25 was assigned to *LFNG*, a candidate gene encoding O-fucosylpeptide 3-beta-N-acetylglucosaminyltransferase. This enzyme has a fucose-specific β-1,3-N-acetylglucosaminyltransferase activity that ensures elongation of O-linked fucose residues. One final significant SNP for 3 Hex 6 HexNAc 1 Fuc on BTA 13 was assigned to *SIGLEC1*, which encodes sialic acid binding Ig-like lectin 1. This enzyme mediates binding of sialic acid to surface proteins and therefore the reason for its association with this neutral OS is currently unclear.

### Other candidate genes in Danish Jersey

Within the very strong and overlapping QTL for LNT and LNH on BTA2, significant SNPs were assigned to two candidate genes involved in glycan synthesis. These include *MAN1C1*, encoding mannosidase alpha class 1C member 1, and PIGV, encoding phosphatidylinositol glycan anchor biosynthesis class V. Mannosidase is a glycosidase involved in the maturation and trimming of N-linked glycans [22], where the core structure contains several mannose residues [1]. *PIGV* encodes a mannosyltransferase involved in involved in the biosynthesis of glycosylphosphatidylinositol (GPI), which is a membrane anchor for e.g. N-linked glycosylation [22]. This again suggests that there could be a close relationship between the free OS and various glycoconjugates. For LNT, significant SNPs were also assigned to *GALNT14* within the detected BTA11 QTL, which is a gene encoding polypeptide N-acetylgalactosaminyltransferase 14. This galactosaminyl transferase catalyses the addition N-acetylgalactosamine at especially serine and threonine positions in proteins and peptides and is thus involved in the formation of O-linked glycans, which have a core structure containing 1 N-acetylgalactosamine [1] linked to target Ser or Thr residues in the protein skeleton. How this relates to LNT is less clear, but again indicates a relation between free OS and glycoconjugates. Further, *COLGAT2*, which encodes collagen beta(1-O)galactosyltransferase 2, was detected as a candidate gene within a QTL on BTA16 for 4 Hex 1 HexNAc. This gene is mainly related to the collagen pathway, but the galactosyltransferase may catalyze the addition of galactose to OS, where e.g. *B4GALT1* encodes the galactosyltansferase being involved in biosynthesis of lactose in the mammary gland [30].

In the present study, specific candidate genes related to OS synthesis have been identified by GWAS in both DH and DJ and high h^2^ are generally reflected in strong SNP associations. Few significant SNPs were common for DH and DJ. However, within breeds the results are expected to be largely transferable, as also documented in a joint GWAS on Danish and Chinese Holstein for milk fatty acid traits, where a large number of overlapping SNPs between populations were identified [31]. Milk OS are recognized for health benefits in relation to gut health by acting as prebiotics and decoys to prevent pathogen binding [20, 32, 33], preventing severe diarrhea [34, 35] and diminish necrotizing enterocolitis in infants [36]. Recent studies have also identified important bioactivities of bovine milk OS that will likely impact adult health, including prevention of diet-induced obesity and its associated increases in intestinal permeability and microbial dysbiosis [37, 38]. The increasing evidence that milk OS promote gastrointestinal health has resulted in optimization of large-scale filtration techniques to recover these compounds from dairy streams [5, 8]. Together with a good potential for selective breeding to increase valuable bioactive OS components in milk, this allows for widespread exploitation of bovine milk OS.

## Conclusion

To our knowledge, this is the first study documenting a solid breeding potential for bovine milk OS and a strong indication of specific candidate genes related to OS synthesis underlying this genetic influence. This new information has the potential to guide breeding strategies to achieve production of milk with a higher diversity and concentration of OS and to ultimately facilitate large-scale manufacture of bovine milk OS.

## Methods

### Sample set

Morning milk samples from 334 DH cows (19 herds) and 300 DJ cows (21 herds) were collected, as outlined by Poulsen et al. [39]. In total, 188 DH sires and 128 DJ sires were represented from the Danish cattle populations. All cows were milked twice a day and fed according to standard practice. The cows sampled were within parity 1 to 3 and all in mid lactation (d 132 to 252).

### OS purification, labeling, and analysis by mass spectrometry

OS were extracted from bovine milk, labeled with isobaric reagents, and multiplexed as described previously by [19]. To measure relative OS abundances, labeled samples were analyzed on an Agilent 6520 Accurate-Mass quadrupole time-of-flight liquid chromatography-mass spectrometry (Nano-LC Chip Q-ToF) system. Instrumental parameters for analysis of labeled OS and methods for extraction of relative OS abundances from the raw data have been described in our previous publication [19]. For the genetic analysis, zeroes were entered for any OS which were below the level of quantification.

### Genotyping and quality parameters

The genotyping was performed as described earlier [28]. In short, genomic DNA was extracted from ear tissue from 334 DH and 300 DJ cows and genotyped with the bovineHD beadchip (www.illumina.com/documents/products/datasheets/datasheet_bovineHD.pdf) [40]. The genotyping was accomplished using an Illumina® Infinium II Multisample assay device. iScan and Beadstudio version 3.1 software was used for scanning and analysis of the SNP chips. Quality parameters for the selection of SNPs were as outlined by [28] and individuals with average GenCall scores below 0.65 were excluded following [41]. The SNP positions were based on the *Bos taurus* genome assembly (ARS-UCD1.2) [42]. Only markers, which were in common between the two breeds were used. This resulted in a total of 494,984 SNP markers. For each gene, the bovine genome location was determined as 5Kb before the start position of the first exon to 5Kb after the end position of the last exon including parts of the upstream and downstream regions of the gene and all introns. A SNP assigned to the corresponding gene when it was located within this region.

### Calculation of the G-matrix

The calculation of the genomic relationship matrix has been described in detail by [28]. The genomic relationship matrix was calculated for each breed separately. A genomic relationship matrix for each chromosome, as described by the first method presented in [43] was calculated.

### Estimation of heritability

The REML approach in DMU was used [44] to estimate the genetic parameters and variance components using the following model in the analysis:

1 $$ \mathrm{Yijkl}=\upmu +\mathrm{herdi}+\mathrm{parityj}+\mathrm{b}1\times \mathrm{DIMk}+\mathrm{animall}+\mathrm{eijkl} $$

Y_ijkl_ represents the phenotype of individual l in herd i and lactation j, μ, herd_i_ (i = 1, 2, …, 19 for DH and i = 1, 2, …, 21 for DJ) and parity_j_ (j = 1, 2, 3 for DH and j = 1,2,3 for DJ) are fixed effects. b_1_ is a regression coefficient for DIM_k_, DIM_k_ is a covariate of days in milk (d132 to d227 for DH, d130 to d252 for DJ), and animal is a random additive genetic effect of animal l based on **G** of animal l [45].

A season effect was not fitted into the model, as the milk samples were collected once on each farm in a short period when the cows were housed indoor.

Heritability estimate was defined as:

2 $$ {\mathrm{h}}^2={\upsigma}_{\mathrm{a}}^2/\left({\upsigma}_{\mathrm{a}}^2+{\upsigma}_{\mathrm{e}}^2\right) $$

where σ^2^_a_ was the genetic variation, and σ^2^_e_ was the residual variation based on univariate analyses.

### Association mapping

The association analysis was performed for each breed separately based on an extension of the linear model 1 with and allele substation effect (b_2_) and a covariate SNP_m_ indicating if a SNP is heterozygote (1), or homozygote (0,2). The effect of the SNP was tested using a Wald test with a null hypothesis of H_0_: b = 0. To correct for multiple testing the false discovery rate (FDR) was used as implemented in the R package “qvalue” version 1.34.0. A SNP was considered significant at an FDR of *P* < 0.10. Proportion of variance explained (PVE) was calculated as:

$$ PVE=\frac{2{\hat{\beta}}^2 MAF\left(1- MAF\right)}{2{\hat{\beta}}^2 MAF\left(1- MAF\right)+\left( se{\left(\hat{\beta}\right)}^22 NMAF\left(1- MAF\right)\right)} $$

Where $\hat{\beta}$ is the estimated SNP effect size, MAF is the minor allele frequency of the SNP, $se\left(\hat{\beta}\right)$ is the standard error of the estimated SNP effect size, and N is the sample size.

## Additional files

Additional file 1: **Table S1.** Significant SNP markers (FDR < 0.10) for milk oligosaccharides for Danish Holstein. (TXT 317 kb)

Additional file 2: **Table S2.** Significant SNP markers (FDR < 0.10) for milk oligosaccharides for Danish Jersey. (TXT 1411 kb)

Additional file 3: **Figure S3.** QQ-plots of P-values for milk oligosaccharides in Danish Holstein. (PDF 713 kb)

Additional file 4: **Figure S4.** QQ-plots of P-values for milk oligosaccharides in Danish Jersey. (PDF 734 kb)
