# Supplementary material for: A genome-wide association study reveals specific transferases as candidate loci for bovine milk oligosaccharides synthesis
Source: BMC Genomics. 2019 May 22;20:404. doi: 10.1186/s12864-019-5786-y (PMC6532250; doi:10.1186/s12864-019-5786-y)
Supplement: Supplementary file 4 — Figure S4. QQ-plots of P-values for milk oligosaccharides in Danish Jersey. (PDF 734 kb) [file 12864_2019_5786_MOESM4_ESM.pdf]

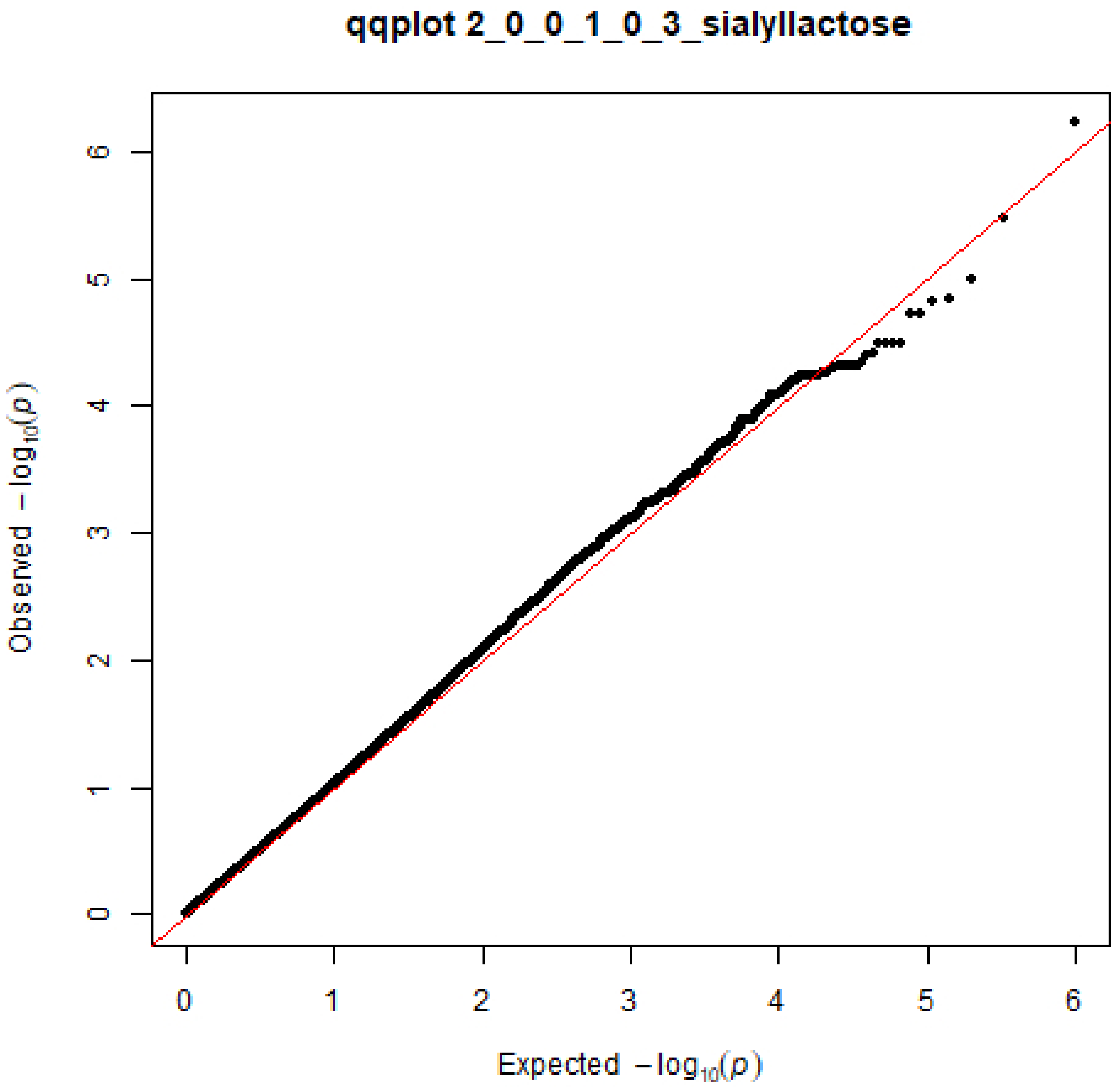
qqplot 2_0_0_1_0_3_sialyllactose
Observed $-\log_{10}(p)$
Expected $-\log_{10}(p)$
0
1
2
3
4
5
6

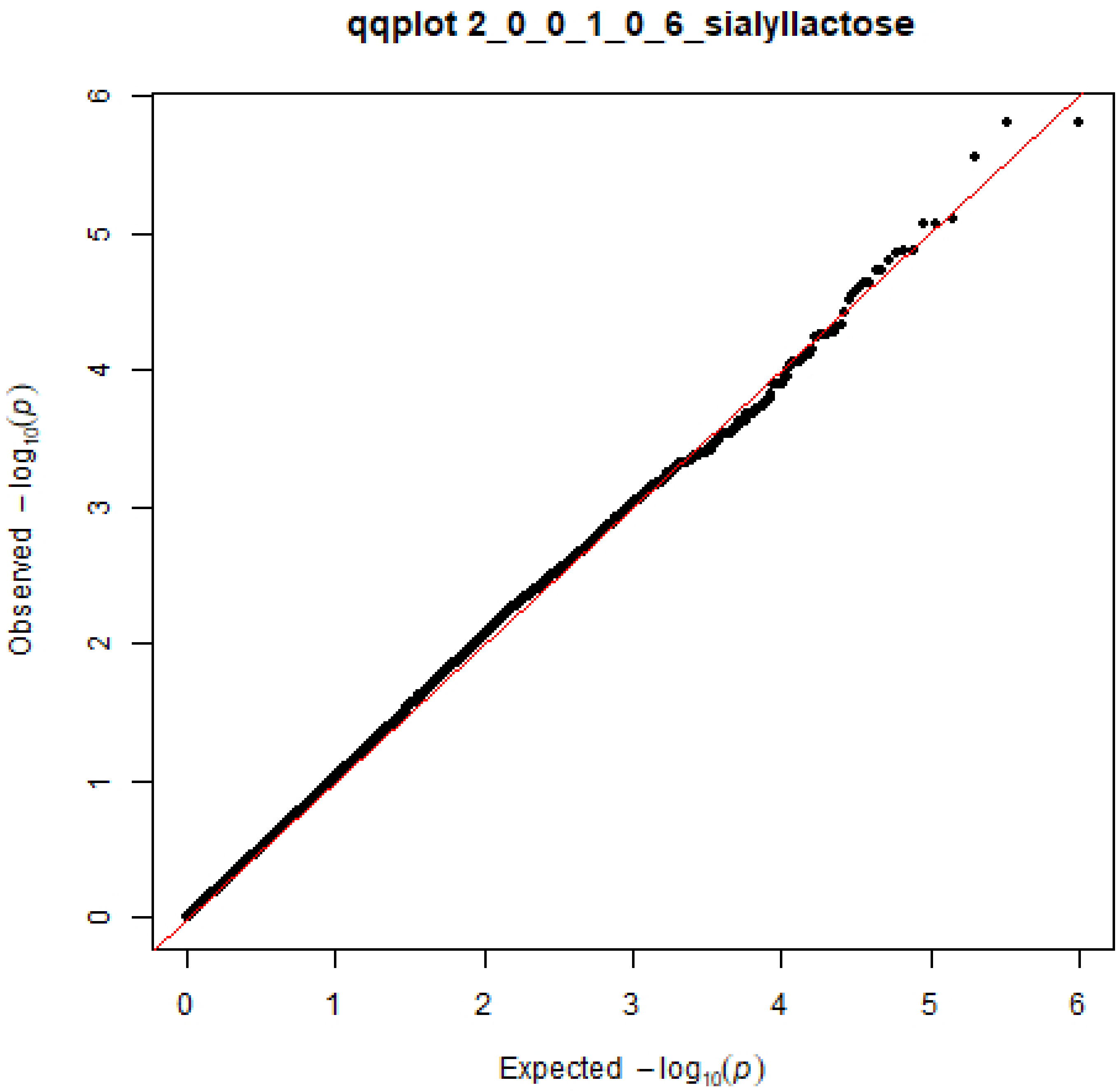
qqplot 2_0_0_1_0_6_sialyllactose
Observed $-\log_{10}(p)$
Expected $-\log_{10}(p)$
0
1
2
3
4
5
6
0
1
2
3
4
5
6

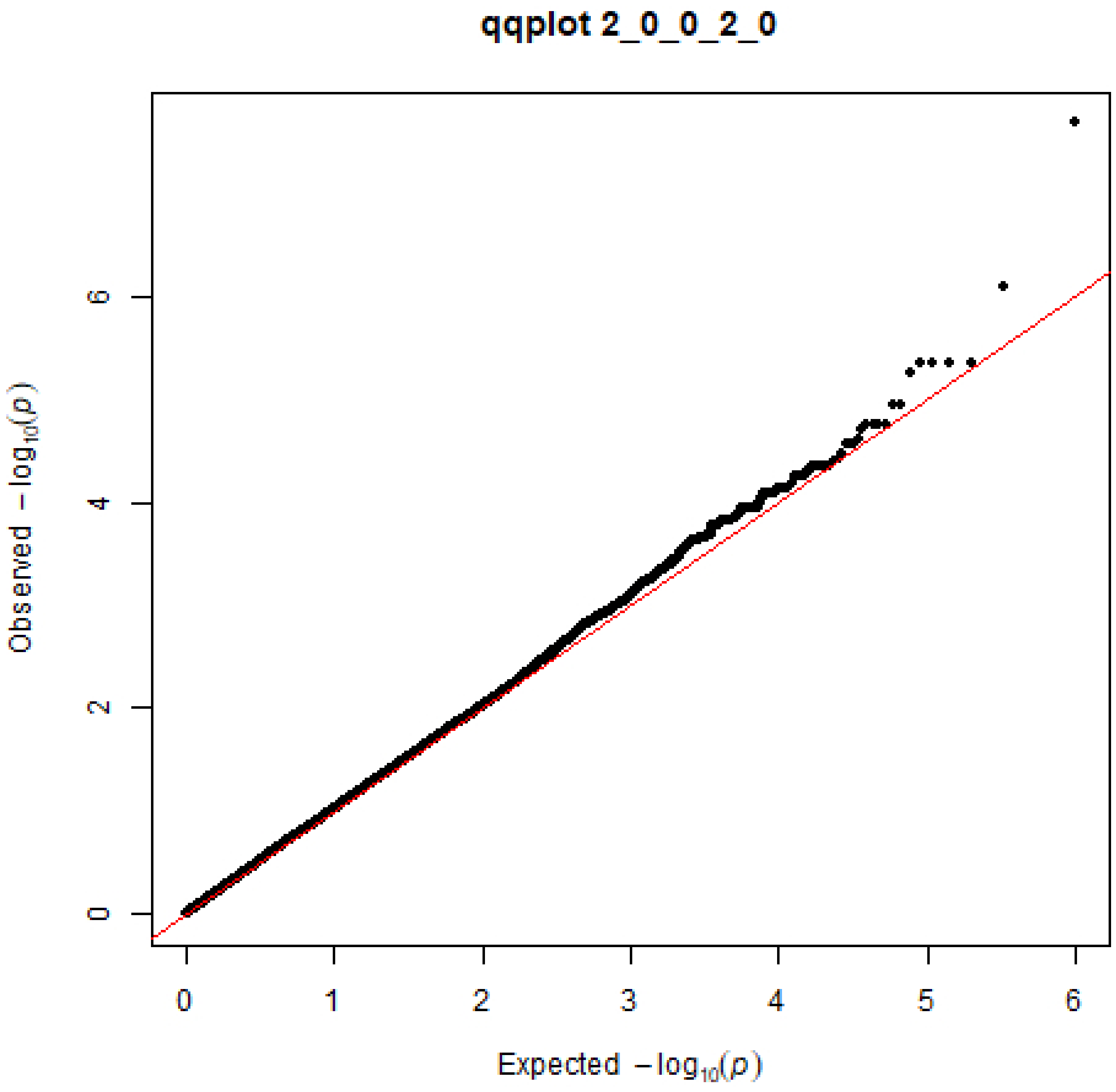
qqplot 2_0_0_2_0
Observed −log10(p)
Expected −log10(p)
0
2
4
6
0
1
2
3
4
5
6

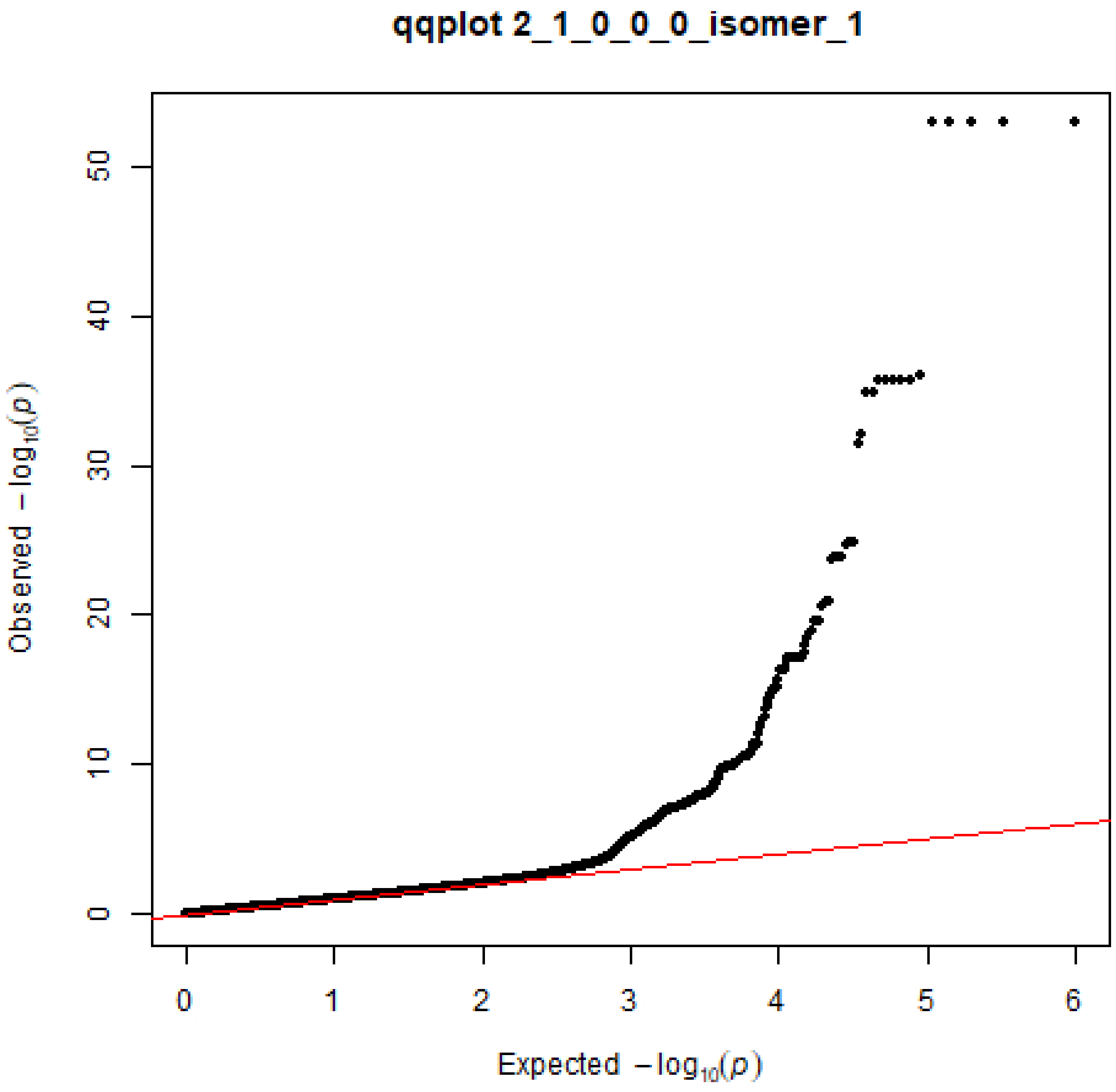

qqplot 2_1_0_0_0_isomer_1
Observed −log10(p)
Expected −log10(p)
0
10
20
30
40
50
0
1
2
3
4
5
6

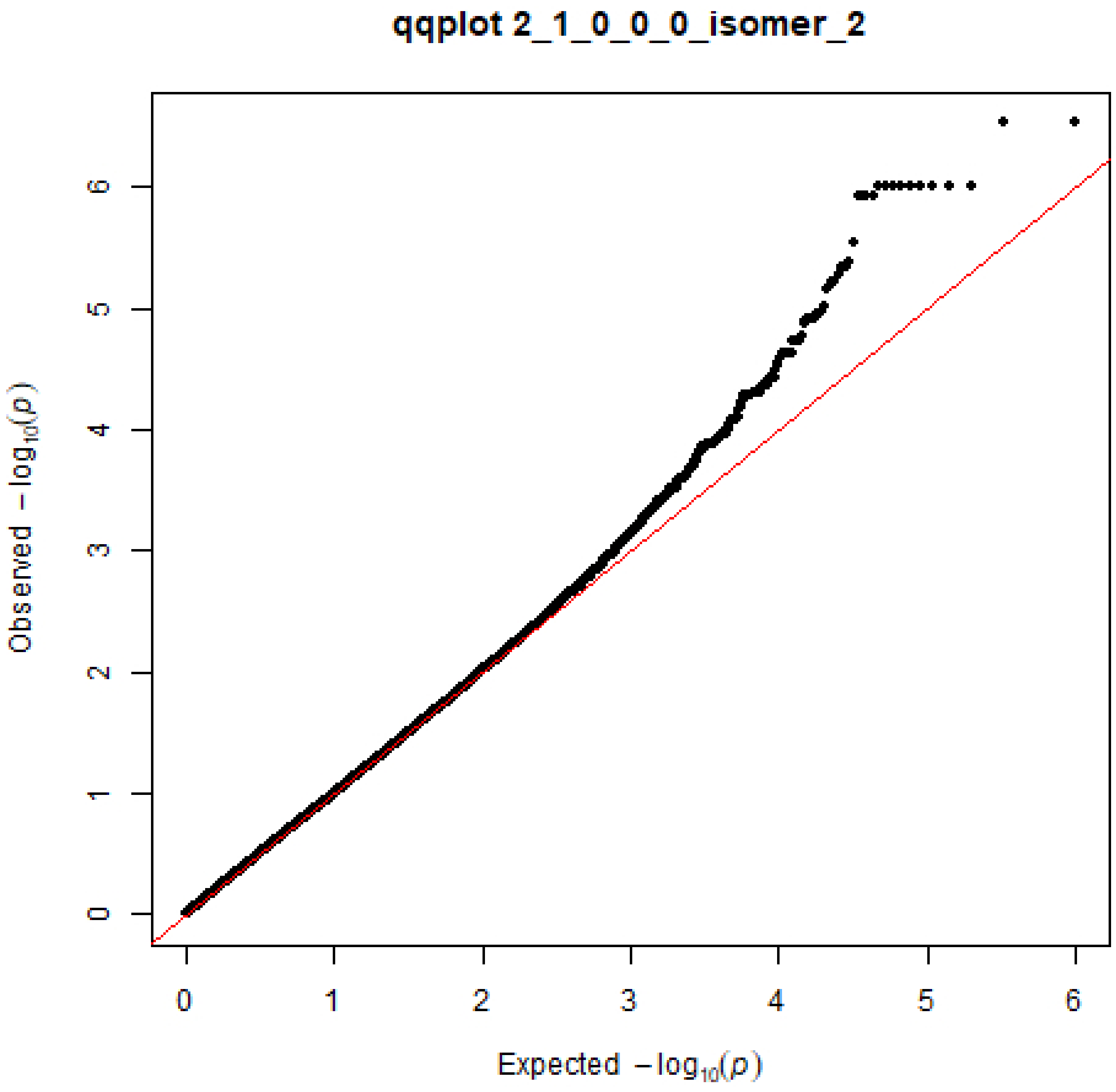
qqplot 2_1_0_0_0_isomer_2
Observed −log10(p)
Expected −log10(p)
0
1
2
3
4
5
6

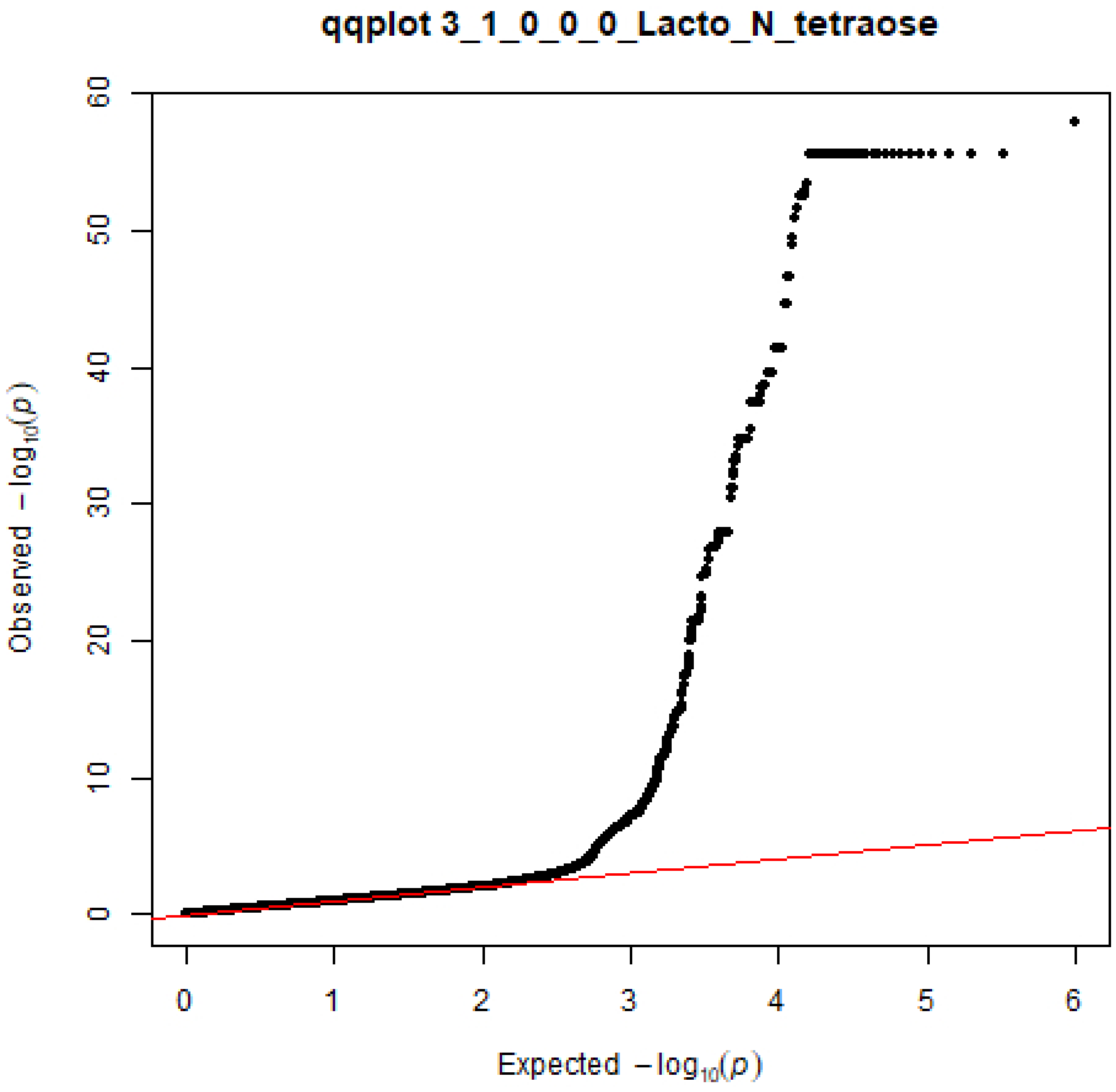

qqplot 3_1_0_0_0_Lacto_N_tetraose
Observed $-\log_{10}(p)$
Expected $-\log_{10}(p)$
0
10
20
30
40
50
60
0
1
2
3
4
5
6

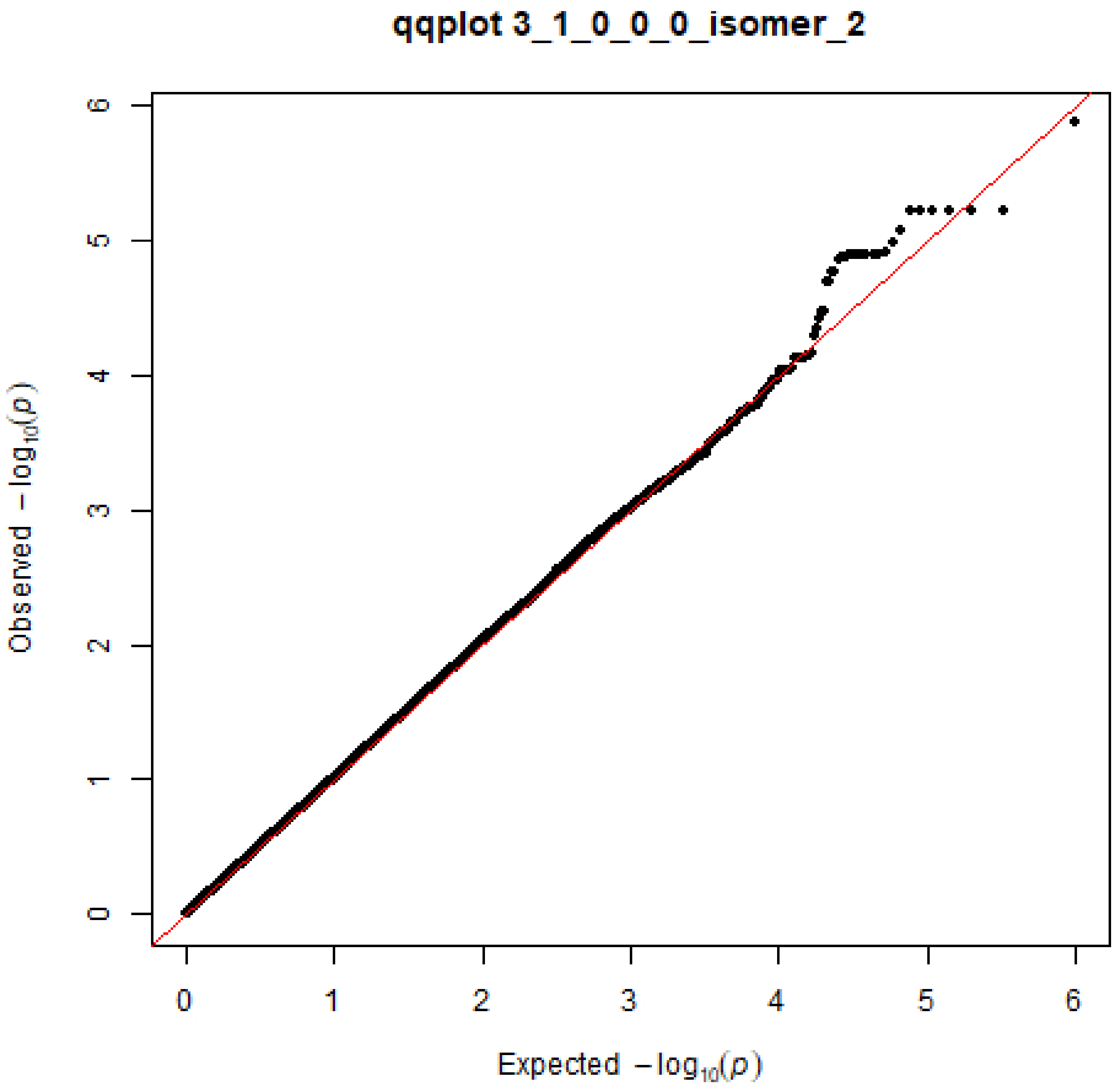

qqplot 3_1_0_0_0_isomer_2
Observed −log10(p)
Expected −log10(p)
0
1
2
3
4
5
6

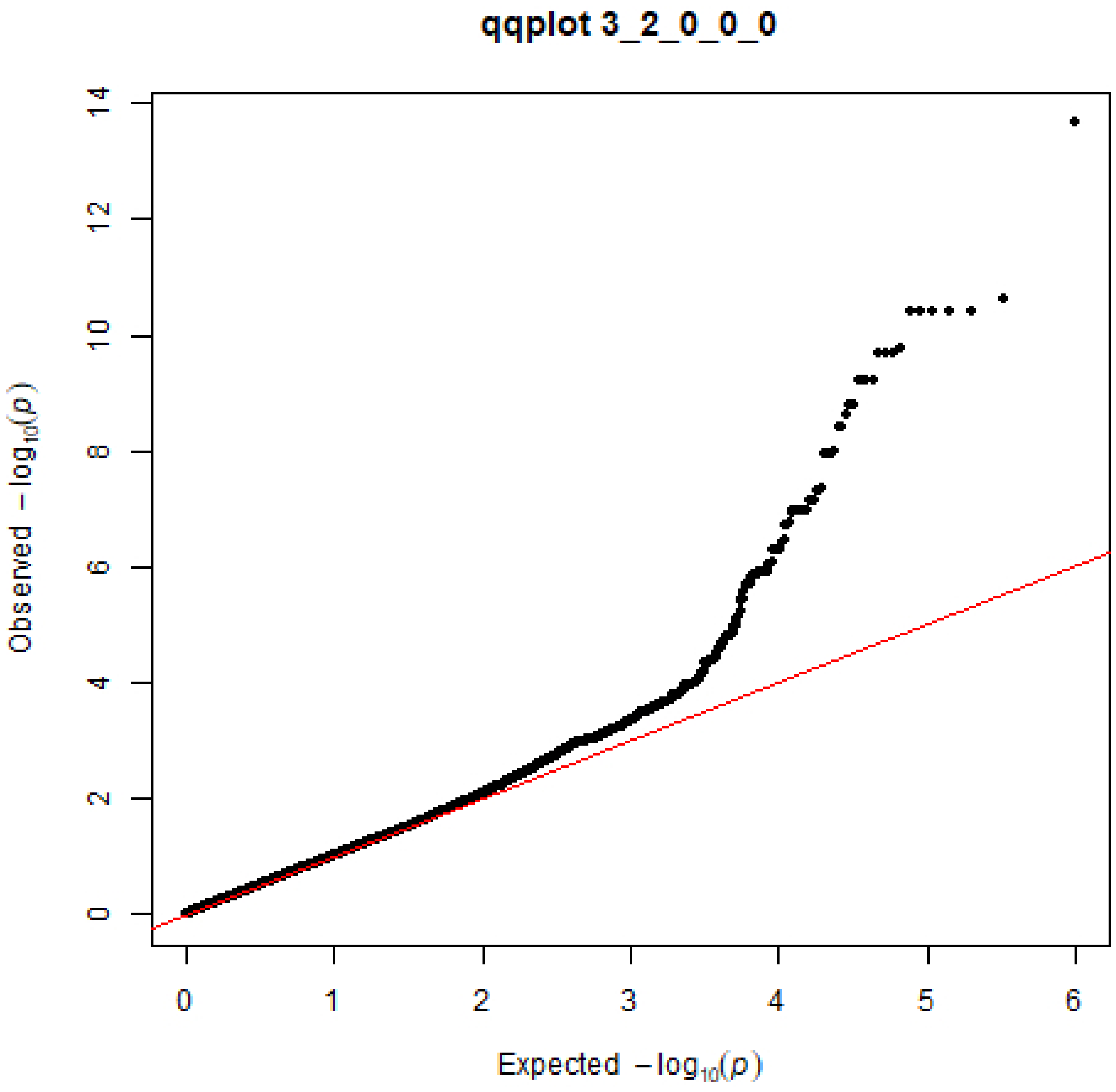
qqplot 3_2_0_0_0
Observed −log10(p)
Expected −log10(p)
0
2
4
6
8
10
12
14
0
1
2
3
4
5
6

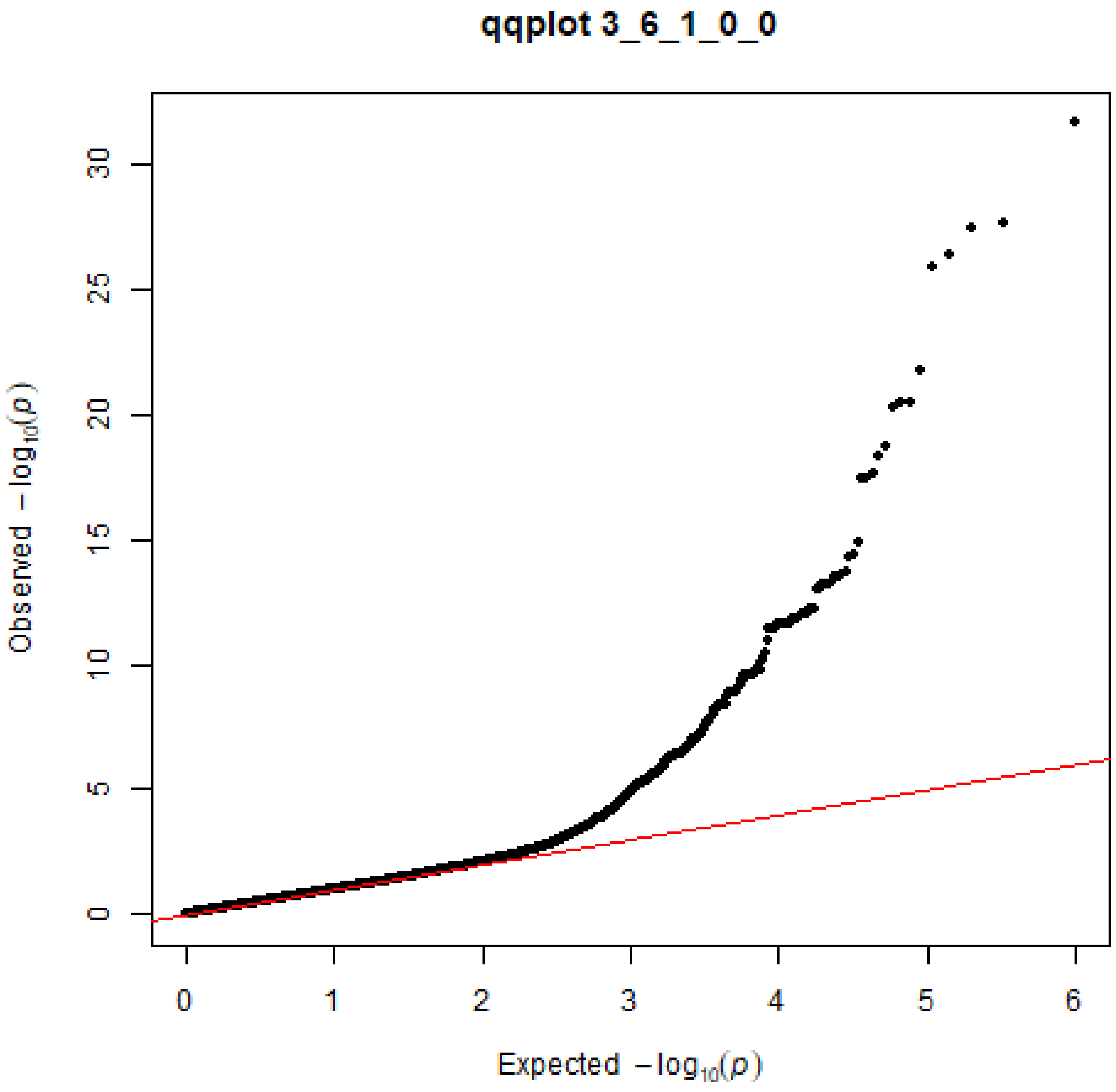

qqplot 3_6_1_0_0
Observed −log10(p)
Expected −log10(p)
0
5
10
15
20
25
30
0
1
2
3
4
5
6

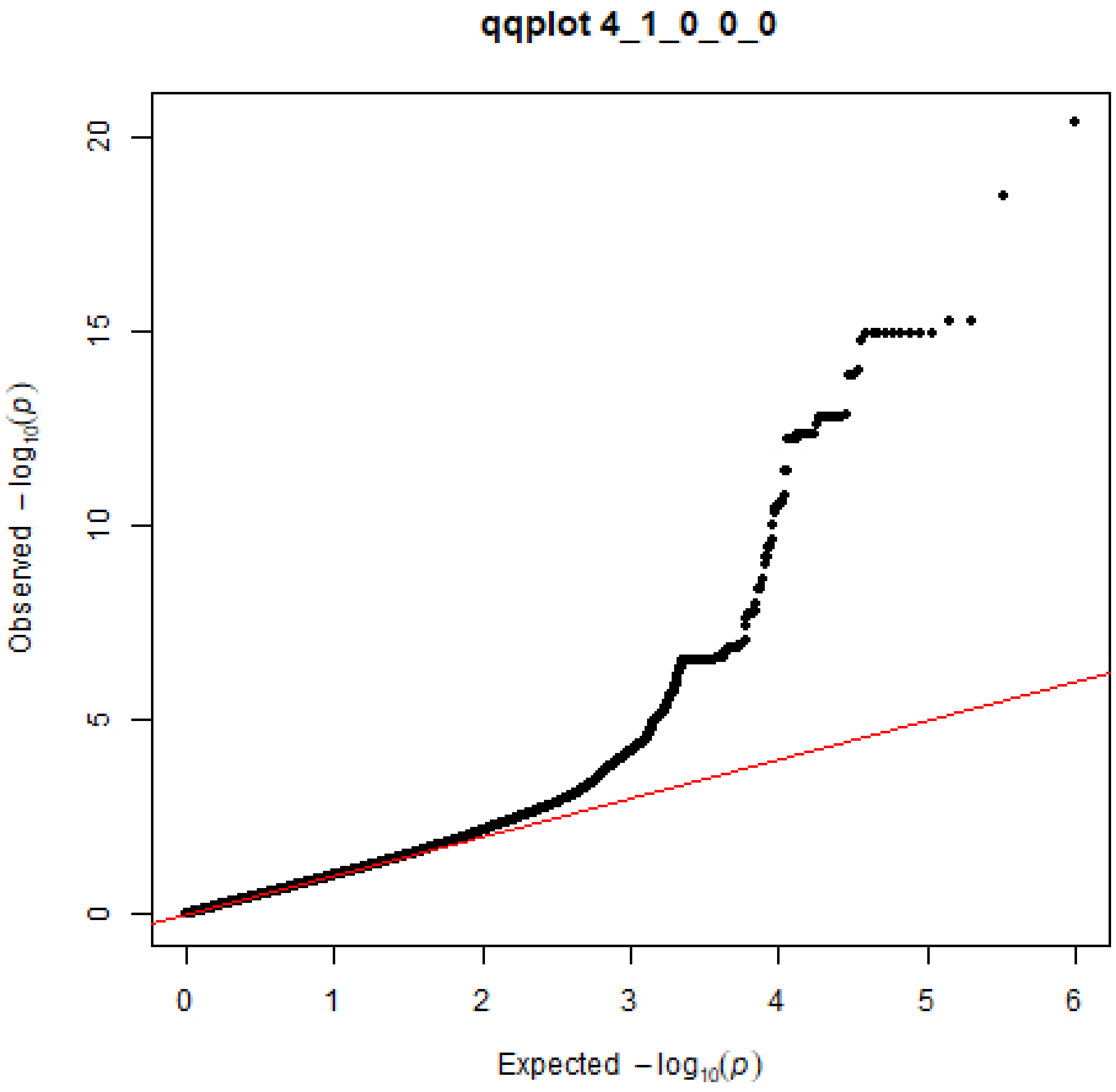
qqplot 4_1_0_0_0
Observed −log10(p)
Expected −log10(p)
0
5
10
15
20
0
1
2
3
4
5
6

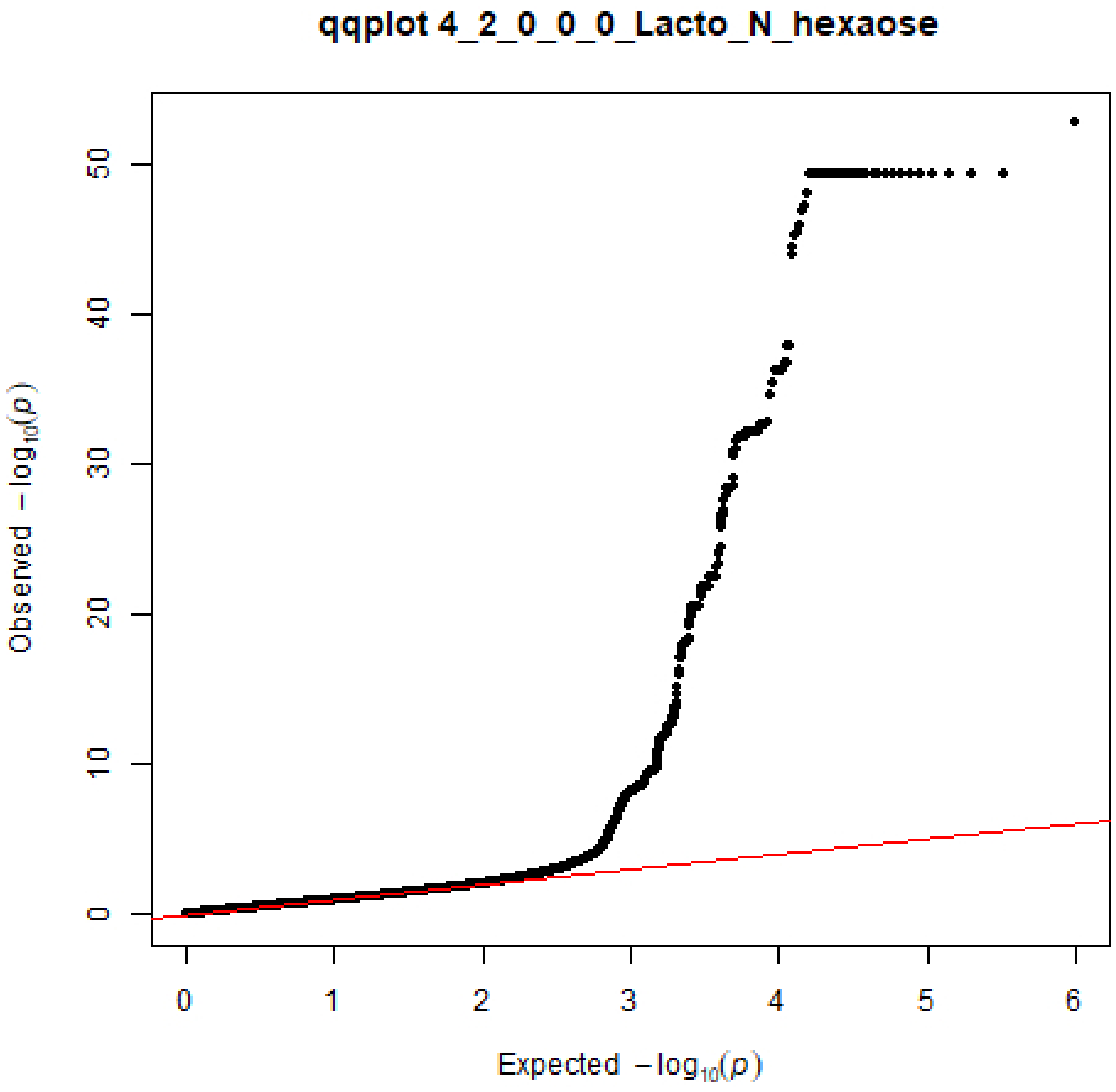
qqplot 4_2_0_0_0_Lacto_N_hexaose
Observed $-\log_{10}(p)$
Expected $-\log_{10}(p)$
0
10
20
30
40
50
0
1
2
3
4
5
6

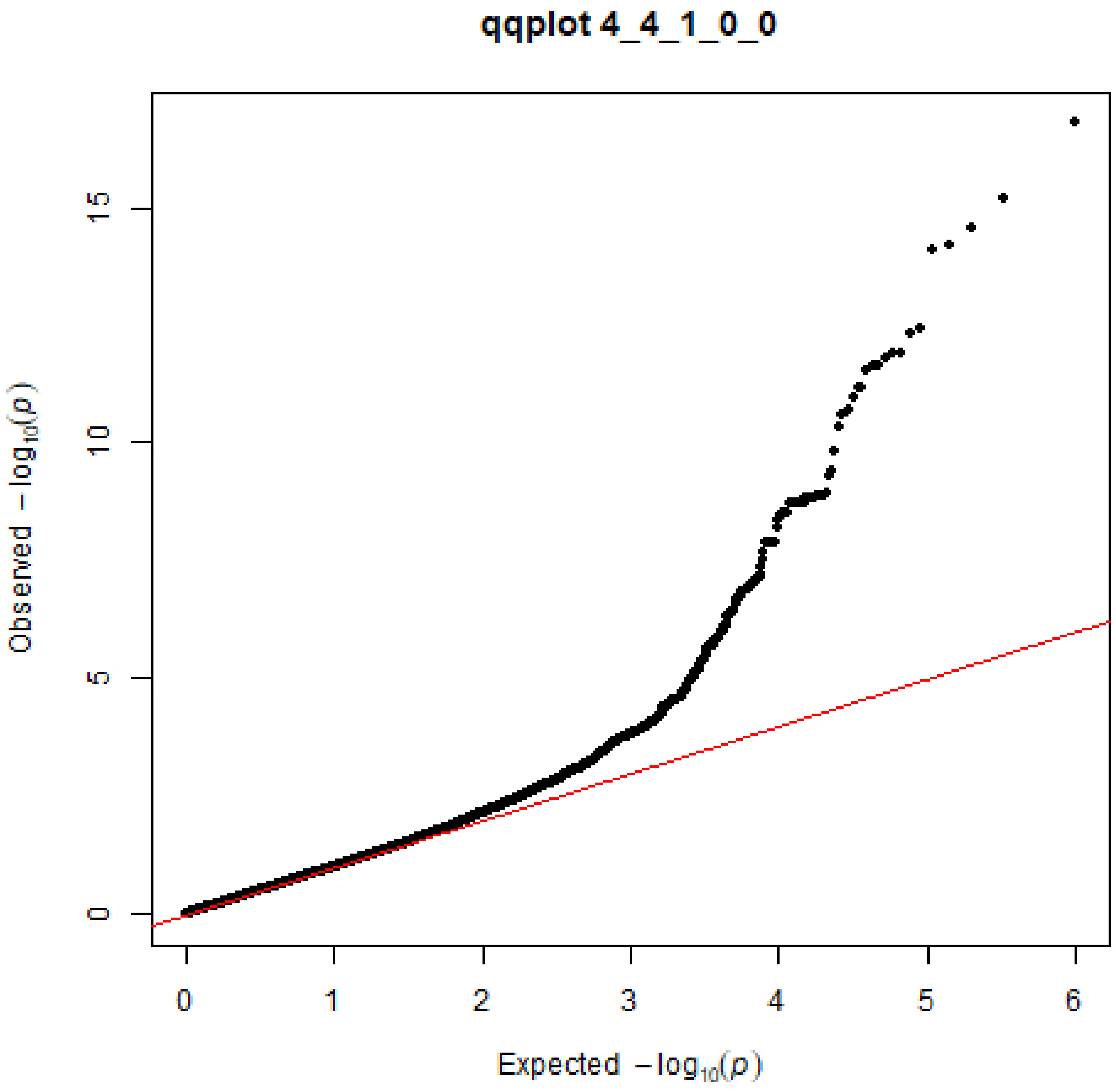
qqplot 4_4_1_0_0
Observed −log10(p)
Expected −log10(p)
0
5
10
15
0
1
2
3
4
5
6

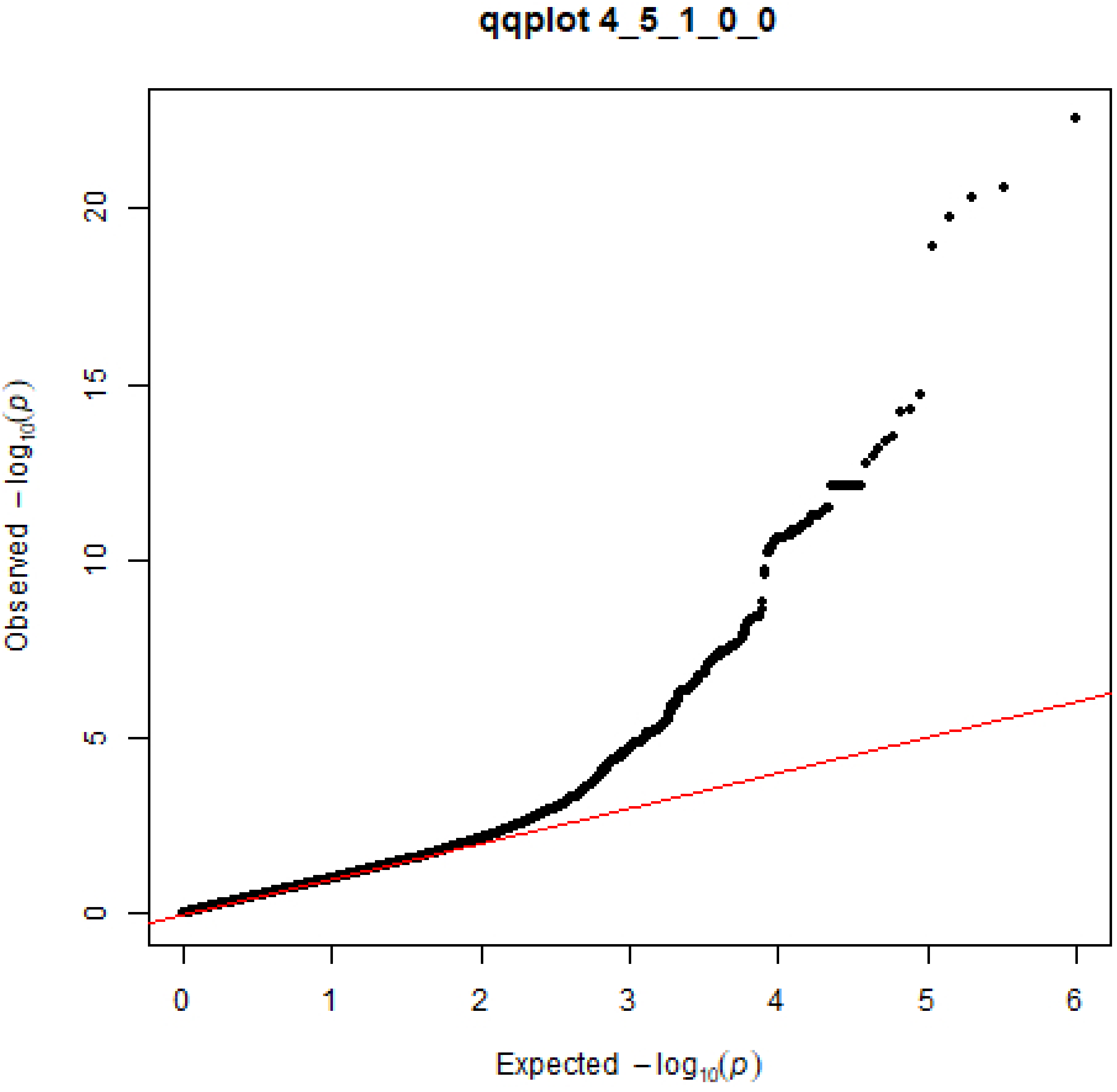
qqplot 4_5_1_0_0
Observed −log10(p)
Expected −log10(p)
0
5
10
15
20
0
1
2
3
4
5
6

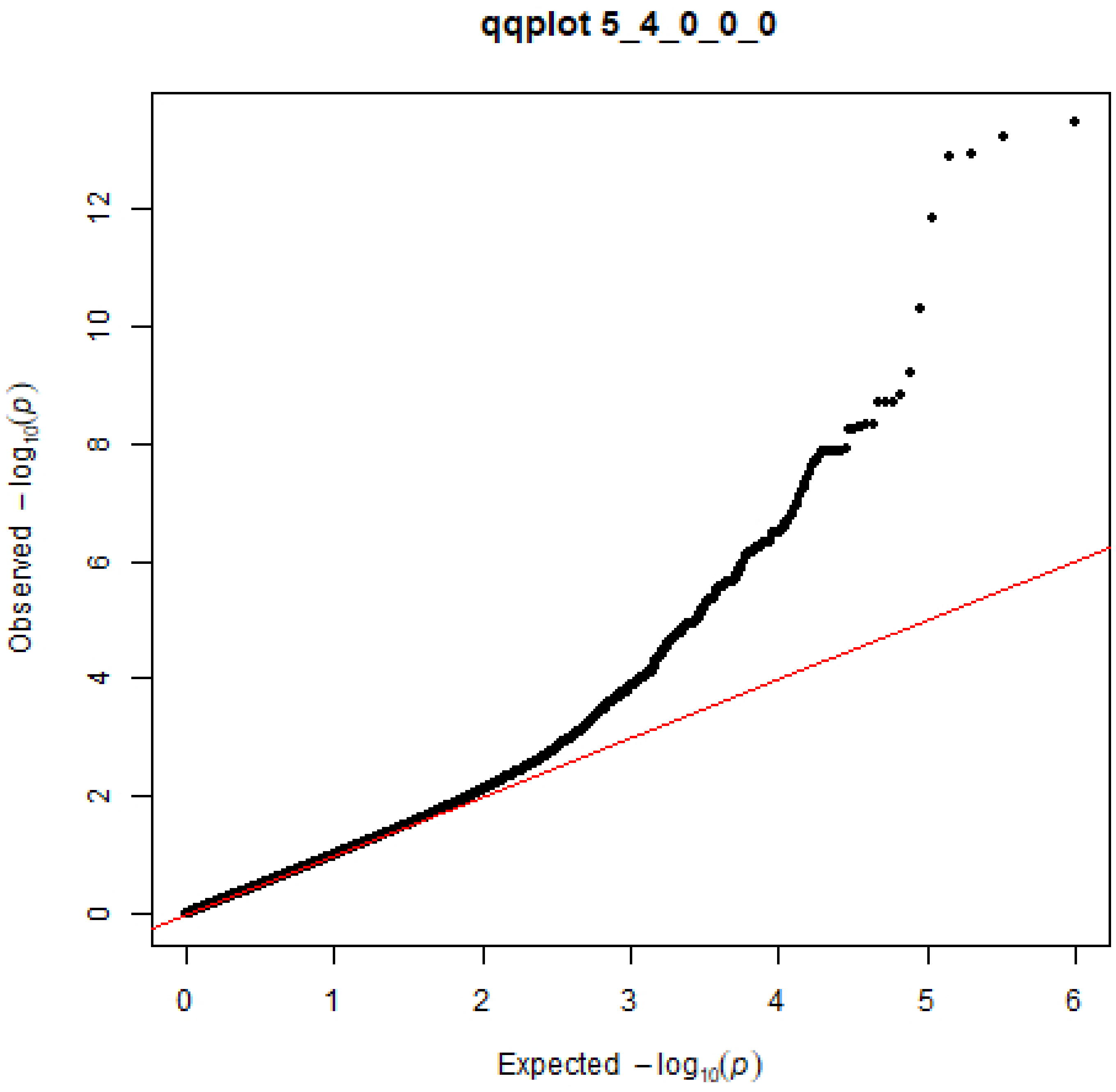
qqplot 5_4_0_0_0
Observed $-\log_{10}(p)$
Expected $-\log_{10}(p)$
0
2
4
6
8
10
12
0
1
2
3
4
5
6

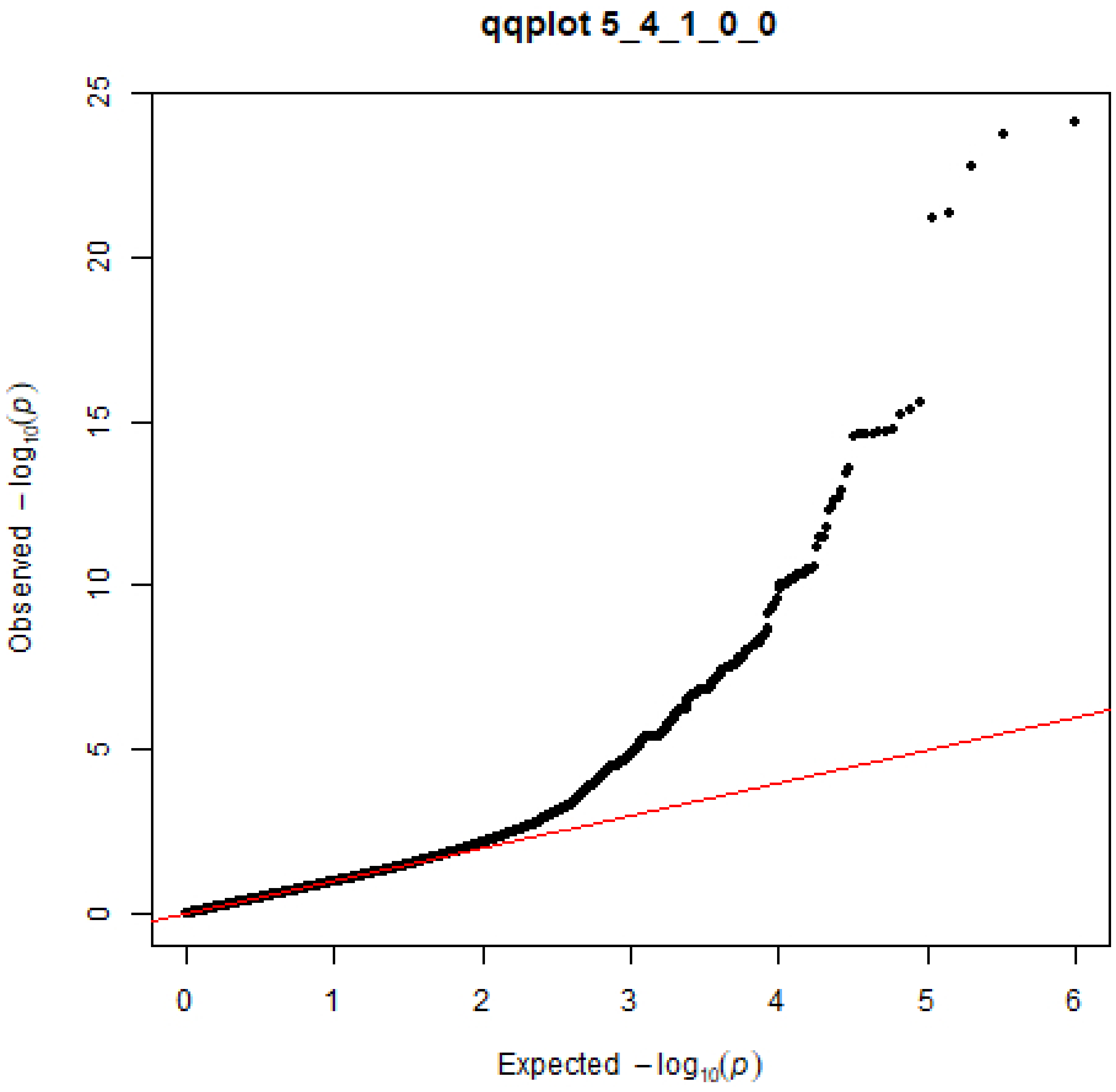

qqplot 5_4_1_0_0
Observed −log10(p)
Expected −log10(p)
0
5
10
15
20
25
0
1
2
3
4
5
6
